# The geography of corporate fake news

**DOI:** 10.1371/journal.pone.0301364

**Published:** 2024-04-17

**Authors:** Alper Darendeli, Aixin Sun, Wee Peng Tay

**Affiliations:** 1 Nanyang Business School, Division of Accounting, Nanyang Technological University, Singapore, Singapore; 2 School of Computer Science and Engineering, Nanyang Technological University, Singapore, Singapore; 3 School of Electrical and Electronic Engineering, Nanyang Technological University, Singapore, Singapore; Rikkyo University, JAPAN

## Abstract

Although a rich academic literature examines the use of fake news by foreign actors for political manipulation, there is limited research on potential foreign intervention in capital markets. To address this gap, we construct a comprehensive database of (negative) fake news regarding U.S. firms by scraping prominent fact-checking sites. We identify the accounts that spread the news on Twitter (now X) and use machine-learning techniques to infer the geographic locations of these fake news spreaders. Our analysis reveals that corporate fake news is more likely than corporate non-fake news to be spread by foreign accounts. At the country level, corporate fake news is more likely to originate from African and Middle Eastern countries and tends to increase during periods of high geopolitical tension. At the firm level, firms operating in uncertain information environments and strategic industries are more likely to be targeted by foreign accounts. Overall, our findings provide initial evidence of foreign-originating misinformation in capital markets and thus have important policy implications.

## 1. Introduction

The last decade has witnessed an unprecedented proliferation of fake news on social media [1]. The 2016 and 2018 U.S. elections demonstrated the vulnerability of domestic politics to false stories originating from foreign countries, and the World Economic Forum (WEF) identifies massive and systematic digital disinformation as one of the top global risks in its 2019 Global Risks Report [2]. A rich academic literature also examines the effects of fake news and foreign interference on politics [3–6]. Yet relatively few papers have considered the role of foreign actors in spreading *corporate* fake news about a country’s firms.

We define “corporate fake news” as negative false information spread about a company and later denied by a credible source (see Table 1 for several examples of corporate fake news) [7]. We focus on the dissemination of fake news on Twitter (now X), since this prominent social media platform lends itself to analyzing economic and financial issues. For example, 71% of the Twitter users in the U.S. have reported getting news on the site, and 48% of institutional investors use social media to “read timely news” [8]. The most popular fake news stories have been widely shared on Twitter [9], spreading farther, faster, more deeply, and more broadly than true news [10]. Research has also shown the use of fake news to manipulate stock prices in capital markets [11]. Indeed, a J. P. Morgan Chase director cites “a combination of domestic political groups, analysts and foreign actors who are amplifying negative headlines to sow discord and erode faith in markets” [12], and a recent article points out the potential role of foreign actors in a disinformation campaign against the Pfizer/Biontech COVID-19 vaccine [13]. Foreign actors can use the growing importance of social media (and the ease with which it can be manipulated) to amplify the effect of fake news on firms, erode confidence in capital markets, and distort efficient resource allocation.

**Table 1:** Examples of corporate fake news.

Company	Category	Publication Date	Title	Claim	URL Link
VICTORIA’S SECRET	Data Privacy	26 August 2020	Victoria’s Secret Bra ‘Tracking Devices’	Victoria’s Secret bras’ tracking devices are part of a sex-trafficking conspiracy.	https://www.truthorfiction.com/victorias-secret-bra-tracking-devices/
NORDSTROM	Other (Financial)	15 February 2017	Nordstrom Files for Chapter 11 After Scott Baio Boycott	Retail chain Nordstrom filed for Chapter 11 bankruptcy after Scott Baio boycotts.	https://www.snopes.com/fact-check/nordstrom-chapter-11-baio-boycott/
YUM BRANDS	Product	01 February 1999	Does KFC Use Mutant Chickens?	The government forced KFC to stop using the word ‘chicken’ in its name because it serves meat derived from mutant animals.	https://www.snopes.com/fact-check/kfc-mutant-chickens/
META	Founder/Executive Management	14 March 2017	Did Mark Zuckerberg Announce His Resignation from Facebook?	Mark Zuckerberg said that he was “disgusted with social media” and that he was leaving Facebook as a result.	https://www.snopes.com/fact-check/mark-zuckerberg-resignation/
WALMART	Religion	10 November 2016	Muslim Woman Told to ‘Hang Herself’ with Hijab at Walmart	A woman pulled a Muslim woman’s hijab inside a Walmart and told her, “Go hang yourself with it around your neck.”	https://www.snopes.com/fact-check/muslim-woman-told-to-hang-herself-with-hijab-at-walmart/
NETFLIX	Founder/Executive Management	14 September 2020	Was the Netflix CEO Arrested on Child Pornography Charges?	Netflix CEO Reed Hastings was arrested on suspicion of possessing child pornography.	https://www.snopes.com/fact-check/netflix-ceo-child-porn-arrest/
**TARGET**	**Politics**	**19 May 2016**	**Target to Discontinue Sales of American Flag**	**Target will no longer sell American flags in its stores.**	**https://www.snopes.com/fact-check/target-american-flag-removal/**
DOLLAR TREE	Operations	20 September 2017	Dollar Tree Closing All Stores?	Dollar General, Family Dollar, or Dollar Tree is closing all stores.	https://www.snopes.com/fact-check/dollar-tree-closing-stores/
MODERNA	Product	26 May 2021	Vaccine Ingredient SM-102 Is Safe	The COVID-19 vaccine from Moderna uses an ingredient called SM-102 to deliver the mRNA that carries instructions for how to develop antibodies against the novel coronavirus. A widely shared video is now spreading the falsehood that SM-102 is harmful.	https://www.factcheck.org/2021/05/scicheck-vaccine-ingredient-sm-102-is-safe/
COCA-COLA	Product	27 February 2001	Can Coca-Cola Dissolve Teeth?	A tooth left in a glass of Coca-Cola will dissolve overnight.	https://www.snopes.com/fact-check/coke-dissolves-teeth/
STARBUCKS	Other (Corporate Social Responsibility)	01 August 2018	Is Starbucks Replacing Plastic Straws with Paper Straws Wrapped in Plastic?	Starbucks is replacing single-use plastic straws with paper straws wrapped in single-use plastic packaging.	https://www.snopes.com/fact-check/starbucks-plastic-straws/
MC DONALD’S	Product	03 September 2014	“McDonald’s hamburgers are only 15 percent ‘real beef.’ The other 85 percent is meat filler cleansed with ammonia, which causes stomach and intestinal cancer.”	Facebook meme claims McDonald’s burgers are made with 85 percent ‘meat filler,’ which causes cancer	https://www.politifact.com/factchecks/2014/sep/03/facebook-posts/blogger-claims-mcdonalds-burgers-are-made-85-perce/

Despite its importance, however, there are few large-scale empirical investigations of corporate fake news and its geographical origins. What are the characteristics of corporate fake news on social media? Do fake news stories pertain only to accounting/financial issues, or are they also related to a wider range of issues such as the politics, products, or operations of a firm? To what extent do these fake rumors circulate on Twitter? And most importantly, what are the geographic locations and characteristics of Twitter users who start the rumors?

In answering these questions, we face two primary challenges: (i) systematically identifying corporate fake news, and (ii) predicting the locations of rumormongers. To tackle the first challenge, we collect a comprehensive sample of (verified) corporate fake and non-fake news from prominent fact-checking organizations (i.e., Snopes.com, Factcheck.org, Politifact.com, and Truthorfiction.com). We automatically scrape the websites and use a mix of automated and manual methods to link the news to related companies. We hire and train human coders to manually match fact-checked news to firms and classify their contents to identify the topics in the text. Our approach allows us to identify not only financial news, but also news that captures other aspects of a firm’s attributes (e.g., religion, founders, products, politics, etc.). We identify 541 (144) corporate fake (non-fake) news stories about 126 (67) unique firms between January 2012 and June 2021. We also identify the source of the fake news stories by following the citation trail and find that 42.51% of the corporate fake news is initially seeded in social media (e.g., Twitter, Facebook), followed by news sites (13.68%). The news also spans a variety of topics, including firms’ politics (37.7%), products (22.6%), operations (16.8%), and founders/executives (6.8%).

To tackle the second challenge, we search mentions of the fact-checked news on Twitter to identify the country locations of users who spread the news. However, the location of Twitter users is hard to pin down, as most users do not voluntarily (or accurately) disclose their location: indeed, they may enter irrelevant text in the data field or intentionally manipulate their locations to obfuscate the origin of their tweets [14]. To overcome this challenge, we employ a machine-learning model to predict the locations of rumormongers on Twitter. We use a comprehensive global sample of around 4 million geo-tagged tweets to train a location-prediction model developed in the computer science literature [15, 16]. We use geo-tags in tweets as reliable ground-truth data because geographic coordinates are appended to tweets based on the location of the mobile device. Specifically, we use tweet text in combination with metadata (i.e., tweet language, user-declared location, user name, and user description) to train a recurrent neural network (RNN) to predict the location of geo-tagged tweets. We concatenate the features in a text, represent them as word embeddings, and use long short-term memory (LSTM), a model that retains longer-range dependencies in text sequences [16, 17]. Although more advanced models exist for text classification tasks in general, given our problem setting, LSTM is one of the most suitable models given its effectiveness and low cost compared to more advanced models. We split our geo-tagged data into training, validation, and test sets, and we evaluate the accuracy of the model in a test set using a variety of model architectures, features, and hyperparameters (see S1 Appendix in S1 File for details). Our fine-tuned model’s predictive accuracy is 88.76%. In other words, we can correctly predict the location of 88.76% of Twitter users’ countries based on their tweets and metadata. This accuracy is comparable to that of other location-prediction models in the literature.

Geotagging, however, requires the consent of users, and only a small proportion of tweets (around 1%) are geo-tagged. Therefore, we use the trained model to infer the locations of Twitter users without geo-tagging data. This approach allows us to infer the home location of all the users in our sample. Of the 685 fact-checked corporate news items, we identify 294 (87) fake (non-fake) stories that disseminate on Twitter. Using the trained model, we find that corporate fake news is more likely than corporate non-fake news to be initiated by non-US (foreign) accounts. The difference between the percentage of fake and non-fake corporate news originating from foreign accounts (37.56% versus 30.84%) is statistically significant (*t* = 2.30, *p*<0.05). The accounts that spread fake news are relatively new and have more followers, and the fake news is retweeted more than the non-fake news. We also compute the percentage of users with bot-like behavior, as bots can be used to disseminate low-credibility information [18]. We find that the users who spread fake news are more likely to exhibit bot-like characteristics (*t* = 36.48, *p*<0.00).

After introducing the data, we present several stylized facts about the geographical origins of fake news (at the country level) and the determinants of a firm being targeted by fake news (at the firm level). At the country level, we measure the percentage of Twitter users in a country who are spreading the fake news and find that fake news is more likely to originate from African and Middle Eastern countries. The top five countries spreading corporate fake news are Oman, Jordan, Morocco, Qatar, and Lebanon. In contrast, non-fake news is most likely to originate from Western countries (e.g., Austria, Finland, Poland, and Denmark). We also use two metrics for distributional analysis of geographical locations. First, we use Kullback-Leibler (KL) divergence as a non-parametric approach to compare the “distance” from distribution of the percentage of fake and non-fake news spread from individual countries. The KL divergence is 0.62 and statistically significant (*SE* = 0.18, 95% CI[0.27,0.97]), suggesting that the set of countries spreading corporate fake news is different from the set of countries spreading non-fake news. Second, we compute median relative polarization (MRP) to compare the concentration of countries spreading fake and non-fake corporate news. We find that MRP is 0.33 and statistically significant (*SE* = 0.11, 95% CI[0.12,0.54]), which suggests that fake news is most likely to originate from a concentrated set of countries. Finally, we show that foreign-originating fake news increases during periods of heightened geopolitical risk.

Second, we estimate firm-level regressions to explore the characteristics of firms targeted by fake news. The results of our tests suggest that two factors can explain part of the variation in exposure to foreign-originating fake news. First, ideological motivations can drive foreign actors to spread misinformation about a country’s firms. The last decade has witnessed a proliferation of cyberattacks by nation-states on the strategic industries of foreign countries. Consistent with this, we find that firms in strategic industries (i.e., the telecommunication, pharmaceutical, semiconductor, computer, and defense industries) are more likely to be targeted by foreign-originating fake news. Second, we find that firms operating in uncertain information environments are more prone to foreign fake news, consistent with information frictions slowing the price-discovery process, which may cause prices to deviate from intrinsic values for prolonged periods of time and create profit opportunities for rumormongers [11].

Our study makes several contributions. First, we contribute to the debate about fake news and misinformation on social media. While research shows that social media is the main conduit through which rumors propagate in the political sphere [7], little work has been done on the geographical origins of rumors in capital markets. By allowing us to infer the geographical locations of rumor starters, our methodology has the potential to inform policymakers on whether foreign influence operations in the political sphere can carry over to the economic domain and capital markets.

Second, we add to the growing literature on information acquisition in the era of social media. Social media platforms can facilitate price discovery by allowing for direct information transfer between firms and consumers/investors [19–23], but the anonymity of users also provides a breeding ground for misinformation and distorts price discovery [24]. Indeed, recent work shows that firms may disseminate truthful negative information about their competitors on social media [25]. In contrast, our focus is on the negative fake news spread about firms on social media.

Finally, location-prediction models have been gaining popularity [15], and researchers have recently used geographic online social networks to estimate population movement patterns [26, 27], forecast economic activity [28], and monitor political events [29], public health [30, 31], the spread of diseases [32], and conspiracy theories [33]. In this work, we use the location-prediction model in the context of fake news dissemination in capital markets.

An important caveat to our study is the descriptive nature of our research design, which may preclude causal interpretation. Nevertheless, it is worthwhile, even at the descriptive level, to conduct a large-scale content analysis of corporate fake news, along with an exploratory analysis of its dissemination and the role of foreign actors on social media. In addition, even if we can identify the foreign origin of a fake news story, attributing it to a foreign state actor or an intentional disinformation operation remains difficult. First, Virtual Private Networks and third-party proxies may mask foreign actors’ identities and locations, making tracking their activity incredibly difficult. Second, the use of vast networks of new and hijacked accounts across multiple platforms complicates attribution as these campaigns adapt and spread rapidly [34, 35] Additionally, platform limitations in data access and analysis capabilities restrict researchers’ ability to pinpoint the source of rumor. Hence, we cannot make any claims about the intent of the users disseminating such news on social media (e.g., whether they are knowingly or unwittingly spreading disinformation, or just joking about the story). While the increased foreign activity during periods of high geopolitical risk and the targeting of strategic industries may provide some clues, it is often difficult to draw conclusions about the originators’ underlying motives. The dynamic nature of social media platforms could also generate some interesting patterns for further exploration. For example, rumormongers may not only initiate rumors but also amplify the fake news (e.g., via retweets or replies to tweets) initiated by another source. It is important to acknowledge that our study focuses solely on the original tweets spreading claims verified by a fact-checking organization. Finally, our dataset includes only rumors that were investigated by fact-checking organizations (and probably excludes less viral rumors), which may lead to a selection bias in the collection of fake news.

## 2. Data and methods

### 2.1. Corporate fake news

We build a comprehensive database of news about U.S. firms that has been debunked by prominent fact-checking organizations (Snopes.com, Factcheck.org, Politifact.com, and Truthorfiction.com). Each fact-checking site has its own classification scheme (e.g., Snopes.com classifies articles into six categories, whereas Politifact.com has nine categories). We normalize the verdicts across different sites by mapping these classes to fake and non-fake news categories (see S2 Appendix in S1 File for details), and we do not include mixed news in the analysis (i.e., news classified as neither true nor false). In doing so, we aim to identify a broad spectrum of corporate fake news over a long time period. Whereas previous studies focus only on the financial information of public companies [11, 36], our approach allows us to identify a comprehensive sample of fake news that targets businesses but is not necessarily financial in nature.

First, we automatically scrape the fact-checking websites to collect all the fact-checked articles and parse the publication date, title, claim, body of the text, and fact-checking ratings for each piece of news. Because a naïve company name keyword search might not fully capture a firm’s products, executives, or subsidiaries (e.g., news about Oreo Cookies may be linked to Mondelez International), we use Named Entity Recognition (NER) (i.e., NLTK, TextBlob, and SpaCy) methods to create a filtered sample of news potentially related to firms. NER methods help us identify an initial subset of news referring to a company (or product) such as KFC, Facebook, or Pfizer or a person such Bill Gates or Steve Jobs. However, because the automated algorithms are trained on non-business textual data, they may generate false positives [37]. Therefore, we manually read the filtered subsample to exclude any non-firm-related news. After reading 100 randomly selected articles, we develop the labeling rules presented in S3 Appendix in S1 File. For example, if the news is about a CEO’s arrest, we include the news in our sample because of its importance to the firm’s operations (see, e.g., https://www.snopes.com/fact-check/italy-bill-gates-arrest/). However, if the news is about a CEO’s private life (or her charitable activities), we do not include it in our sample (see, e.g., https://www.snopes.com/fact-check/bill-gates-planned-parenthood/ or https://www.politifact.com/factchecks/2020/may/14/facebook-posts/no-evidence-gates-foundation-will-profit-coronavir/). We also exclude conspiracy theories, satire, and false statements by politicians [7] and keep only the negative-sentiment news, using the sentiment score determined by the word list in [37] (e.g., we classify news as negative if it contains more negative than positive words and manually read the news to determine the sentiment if the difference between positive and negative words does not exceed 1% of total words).

Second, we manually classify news content to identify the specific topics in the text. We prefer manual annotation (instead of automated annotation algorithms) to develop a deeper understanding of the features of the text given the high level of domain expertise required in our setting. To do this, we start by determining categories based on 50 randomly selected subsets of news. Then, we train three research assistants to annotate the same text independently and compare results. After ensuring the degree of consensus regarding the topics, research assistants continue to independently annotate a larger set of news and identify major categories of news topics. When the research assistants disagree (on a new topic category), the authors discuss the news before reaching an agreement. This way, we identify a broad range of topics including firms’ products and services, operations, data privacy, politics, and founders and executive management. Table 1 shows several examples of corporate fake news classified into the various topical categories.

Finally, we identify the origin of a story by relying primarily on URL links mentioned in fact-checking articles. Fact-checking articles often discuss and cite the source of a claim while debunking the claim. We manually read each URL link in fact-checked articles to determine the origin of the claim and identify its publication date. If there is more than one relevant source, we keep the earliest published source.

### 2.2. Twitter data

We collect historical tweet- and user-level data about corporate news using Twitter Academic API. The API grants academics full access to historical tweets dating back to 2006 (except for deleted tweets and accounts). We identify the original (or source) tweets spreading fake and non-fake corporate news in two steps. First, we collect all the tweets that contain a link to a fact-checking website that evaluates the veracity of a corporate news story. We exclude the original tweets containing a link to a fact-checking website, because our goal is to identify the spread of unverified and contested information, not information verified by fact-checking organizations. The remaining tweets are replies to an original tweet or replies to replies. We also remove tweets that do not directly reply to an original tweet to ensure that a reply containing a link to a fact-checking website is in fact addressing the original tweet.

Second, we extract the URL links to external articles mentioned in the above original tweets (spreading fake and non-fake news) or the URL links mentioned in the fact-checking articles. After manually reading the extracted articles, we identify the URL links about the (fake and non-fake) news. We then extract the original tweets containing a link to any of these articles (which are not necessarily fact-checked through replies). In our search, we transform links to canonical URLs by removing http://, https:// and analytic tracking parameters (i.e., Urchin Tracking Module parameters), and we transform short URLs to the expanded form (to merge different links referring to the same article). Through this process, we identify a sample of original tweets mentioning a fact-checked corporate news story on Twitter. We define the sender of the original tweet (i.e., the source of the Twitter cascade) as the rumor source.

These sample filters leave us with 342,818 original tweets (i.e., no retweets or replies) mentioning corporate news verified by a fact-checking organization. Tweet-level variables include the tweet text, timestamp, tweet language, and number of retweets. We also separately query for author-level data—user name, user description, the number of followers, the number of followees, and account creation date—for each unique Twitter user authoring one of the collected tweets. We obtain data for 189,158 unique Twitter users. Finally, we manually match Twitter data to other academic datasets (e.g., COMPUSTAT) using company names. Our code and dataset are available at https://github.com/alperdarendeli/corporatefakenews. The collection and analysis methods comply with the terms and conditions for the source of the data. Our study was also reviewed and approved by the Institutional Review Board of Nanyang Technological University (IRB-2022-349).

### 2.3. Twitter location-prediction model

The geolocation of a rumor source on social media is hard to pin down, either because users do not disclose their home location or do not enter data that correspond to their actual locations [14]. For example, users frequently enter fake locations or sarcastic comments in their profiles (e.g., Jupiter, Outta Space, Out of this world, etc.), making it difficult to infer user location solely from self-declared profile data. The profile locations could also be inaccurate because individuals choose not to publicly share their country of location or intentionally obfuscate the origin of their tweets, as is frequently the case with user-generated input. Hence, location information on Twitter is often far from complete and reliable. To tackle this issue, we employ an LTSM model for location prediction, which is particularly suitable for processing reasonably long and sequential text data [15].

The LSTM model is a type of RNN that can utilize information further back in a sequential chain. To do this, LSTM models use mechanisms called gates that regulate the flow of information being passed from one step to the next. Unlike a vanilla RNN, LSTM includes a forget gate that determines the sort of information that is passed across the sequence. The operations within an LSTM allow the model to keep relevant information (and forget irrelevant information) from the previous steps no matter what the length of the sequence is. Thus, LSTM models do not suffer from the vanishing gradient problem (i.e., the short-term memory problem). This makes LSTM suitable for processing sequential data with long-term dependencies (such as text). We use the model to predict the geographic location of Twitter users [16].

For prediction, we use tweet content in combination with user metadata because the recent literature shows that the metadata can contribute substantially to predictive accuracy and provide valuable location signals [15]. We use a large sample of English and non-English geolocated tweets between 2014 and 2019 as our labeled dataset. We use the locations in geo-tagged tweets as our ground-truth because they are based on the GPS coordinates of mobile devices, which are reliable and difficult to manipulate. The training data consist of 3,927,563 geotagged tweets covering 149 countries and 2,187 cities (see S1 Appendix in S1 File for details).

First, we extract the following text features from the geotagged tweets: tweet text, tweet language, user-declared location, user description, and user name. Other features such as time zone, UTC offset, URL links, and messenger source (e.g., iPhone or Android) can also help predict locations. Time zone and UTC offset, however, cannot be extracted from Twitter API at the time of our study (https://twittercommunity.com/t/upcoming-changes-to-the-developer-platform/104603). The previous literature also shows the limited benefit of URL links and messenger source for the prediction task [16]. We then clean the text by (i) removing links, user name, punctuation, and extra spaces, (ii) separating emoticons, (iii) making all text lower case, and (iv) concatenating the tweet features. We concatenate as follows, inserting special tokens at the front of each text field. A [BLANK] token is also inserted if the specific text field is blank.

‘[TEXT] <cleaned_text> [LANG] <tweet_lang> [LOC] <cleaned_user_declared_location> [DESC] <cleaned_user_description> [NAME] <cleaned_user_name>‘

We then feed the concatenated text into an LSTM model to predict geographic locations. By and large, we follow the model architecture in [16] and pose the geolocation-prediction task as a multi-label classification task of 2,187 cities. In the model, the concatenated text is represented as word embeddings because it better captures words with similar locational semantics in low-dimensional vectors (and produces a more efficient representation of words than one-hot encoding). After transforming the features into machine-readable vectors, we train an LSTM model with a prediction layer. We split 80% of the data into training, 10% into validation, and 10% into test sets. We use stratified sampling to ensure that the geographical distribution of each set is approximately equal. The model learns a weight (or coefficient) for each tweet feature and uses Adam optimization to minimize cross-entropy loss over all possible weights. We fine-tune the model using different hyperparameters on the validation set. Table 2, Panel A reports the selected model hyperparameters. S1 Appendix in S1 File provides technical details about data pre-processing, parameter tuning, and model training steps. The model predicts the geographic location of a tweet at the city level (after which we map the prediction to the related countries). We do not use city-level predictions because the predictive accuracy at the city level is much lower than the accuracy at the country level (51.4% versus 88.8%). And, more importantly, our research question, which examines the prevalence of fake news from foreign accounts, is cast at the country level.

**Table 2:** Twitter location-prediction model.

**Panel A.** Model hyperparameters	
	*Parameters*
*Learning Rate*	0.001 with step decay
*Number of Epochs*	5
*Algorithm*	Adam Optimizer
*LSTM Layers*	2
*Embedding Dimension*	100
*Training*, *Validation and Testing Sets*	{0.80, 0.10, 0.10}
*Sampling Choice*	Stratified
**Panel B.** Predictor power of Twitter features	
*Features*	*Accuracy*
*Majority Predictor*	20.98
*Tweet Language*	39.62
*User Description*	52.13
*User Name*	53.57
*User-declared Location*	66.64
*Tweet Text*	72.48
*Tweet Text + Tweet Language*	72.26
*Tweet Text + User Name*	77.10
*Tweet Text + User Description*	78.08
*Tweet Text + User-declared Location*	86.34
*Tweet Text + User-declared Location + User Description*	87.54
*Tweet Text + User-declared Location + User Name*	87.63
*Tweet Text + User-declared Location + User Description + User Name*	88.41
*All Features*	88.76

This table reports the selected model hyperparameters and the contribution of individual features to predictive accuracy. In Panel A, we show the selected hyperparameters of the model based on a validation set (see S1 Appendix in S1 File for details). In Panel B, we show the accuracy of the model using different combinations of tweet features. Accuracy is the proportion of model predictions that correctly predict ground-truth country location in geotagged tweets. We measure accuracy using an (out-of-sample) test set.

We then evaluate the accuracy of the model at the country level using the test set.

The individual performance of different features is presented in Table 2, Panel B. If we had always predicted that the United States would be the most frequently observed country in the training data, our prediction would be 21% correct. As a simple baseline, we assess our model’s performance against this majority predictor. We start our bottom-up analysis with individual tweet features. We find that tweet text is the most relevant feature for location prediction. Using the text alone, we can correctly predict the location of 72.48% of all tweets. Consistent with the prior literature, we find that augmenting text with user metadata improves accuracy. For example, combining text with user-declared location improves accuracy to 86.34%. When we concatenate all features (tweet text, user-declared location, user description, and user name), the model achieves the best predictive accuracy of 88.76%.

**Fig 1:**
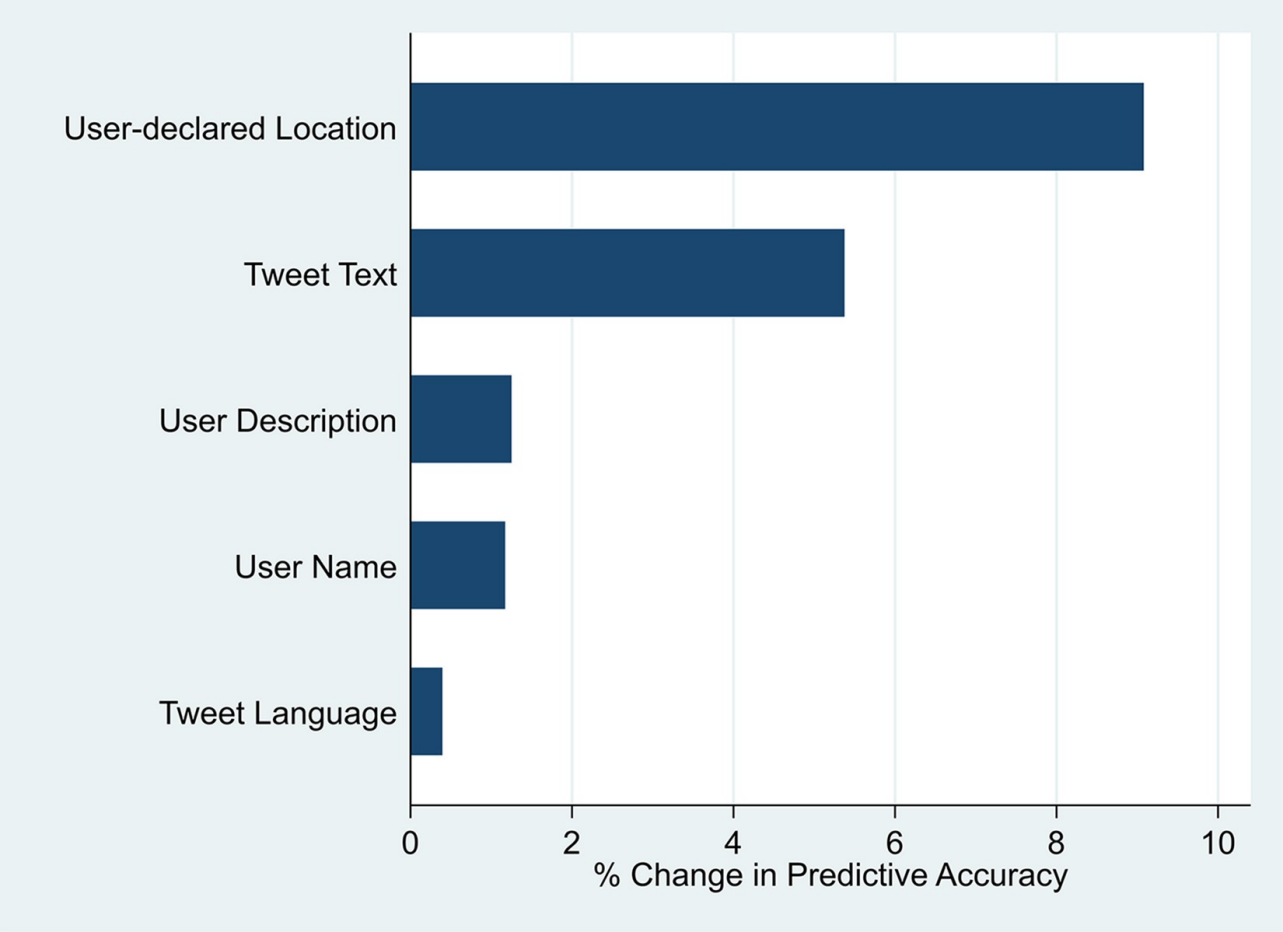
Feature importance analysis. This figure ranks the features of the location-prediction model in terms of their contribution to model performance. For the feature importance analysis, we use the following equation: $\%\Delta \;Predictive\;{Accuracy}_{k}=\frac{(Predictive\;{Accuracy}_{All}-Predictive\;{Accuracy}_{All\backslash \{k\}})\;}{Predictive\;{Accuracy}_{All}}$ The numerator of the equation calculates the decrease in predictive accuracy when a feature is excluded from the predictor set. We then scale the decrease in predictive accuracy by the predictive accuracy of the model when all features are used. The reported numbers are in percentage points.

Next, we perform a leave-one-out feature importance analysis to rank the factors in terms of their contribution to model performance. The analysis relies on the idea that if a feature is not important, excluding it from the predictor set should not noticeably decrease the model’s out-of-sample performance. We iteratively remove one feature at a time and evaluate accuracy. Specifically, we calculate the decrease in the predictive accuracy when a feature is excluded from the predictor set. We then scale the decrease by the predictive accuracy when all predictors are used. Surprisingly, while tweet text separately has the largest influence on the prediction of user locations, we find, in Fig 1, that user-declared location is the top explanatory predictor in the feature importance analysis. This implies that user-declared location may not carry a large weight on its own, but combining it with other features might help improve the predictive accuracy of the model.

Overall, our model significantly outperforms a naïve majority predictor in predicting the geographic locations of tweets. Our model accuracy is also comparable to that of other location-prediction models in the literature. For example, a maximum entropy classifier [38] predicts the country origins of tweets with 88.90% accuracy using tweet language, user-declared location, user language, time zone, offset, user name, user description, and tweet text. A linear classifier model [39] predicts the country origins of tweets with 87.34% accuracy using tweet text, profile location, time zone, and time (in UTC time). Our model’s predictive accuracy is comparable despite our inability to use additional features such as time zone, offset, and user language, which were not accessible via Twitter API at the time of this study.

**Fig 2:**
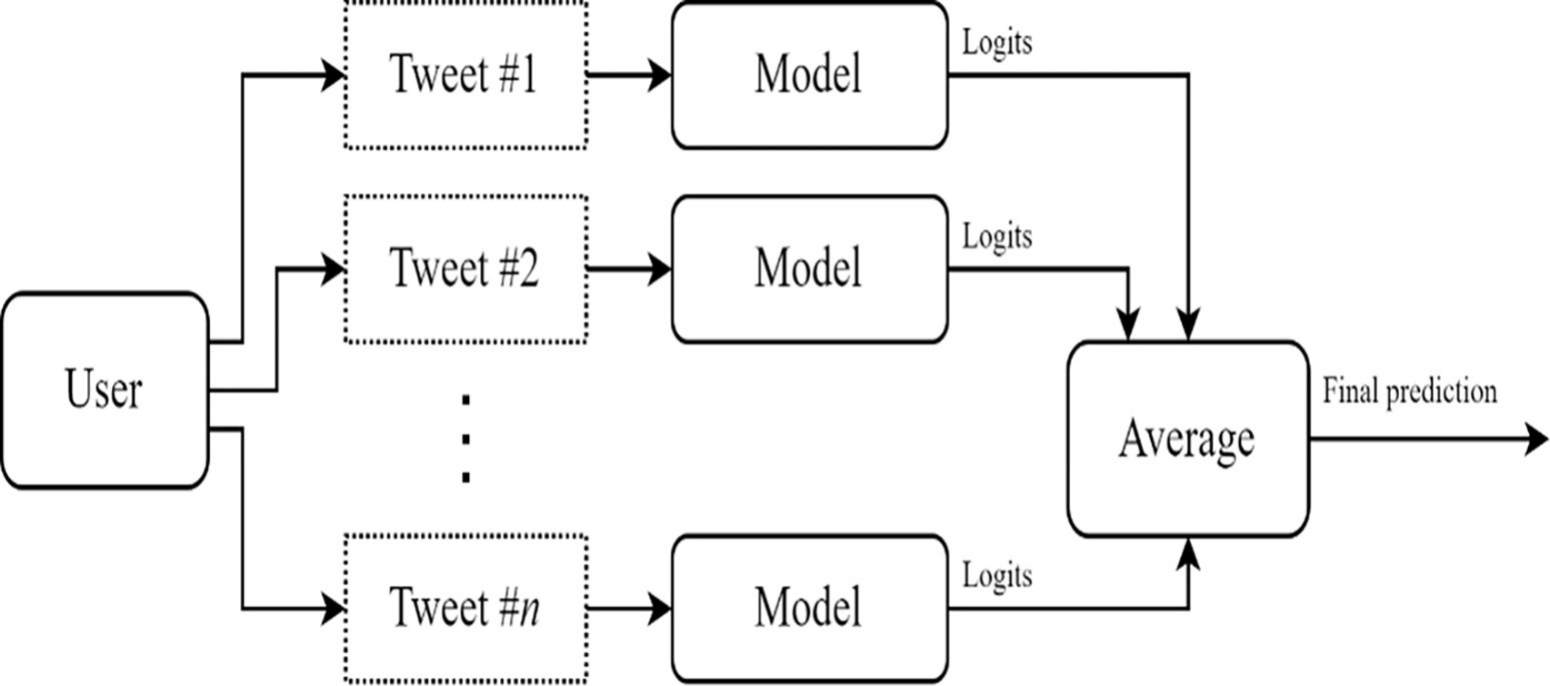
Twitter user location-prediction model. This figure illustrates the location prediction of a Twitter user. We implement a two-step procedure. First, we use our model to predict locations at the tweet level. Specifically, we apply our fine-tuned model’s weights to produce a score (or multiclass logit) for each tweet location. Second, we take the average probabilities of tweet location at the user level to predict the country with the highest score as the location of the Twitter user.

As the authors of [38] show that geo-located and non-geolocated tweets have similar characteristics, we can use a model trained on geolocated tweets to predict the locations of users without geo-tagged data. Therefore, we use our fine-tuned model to predict the locations of tweets disseminating fact-checked corporate news. Fig 2 illustrates the location-prediction procedure at the user level. We use metadata and multiple tweets of each user to predict the user’s country location.

We apply the fine-tuned model’s weights to our sample to produce a score for each tweet’s location. Specifically, we use the softmax function to convert these weights to multiclass logits (or probabilities) for each city and country. For each tweet, the model generates a vector of probabilities Pr(*Cu*_*k*_) = {pr(*C*_*1uk*_), pr(*C*_*2uk*_),…, pr(*C*_*Luk*_)}, where pr(*C*_*1uk*_) is the probability that the user *u*_*k*_ will be assigned to country 1, and so on. Then, we take the average probabilities of tweet locations at the user level and predict that the Twitter user is located in the country with the highest score.

## 3. Results and discussion

### 3.1 Empirical construct

We use the model’s predictions to construct *Foreign Corporate Fake News (%)* as the key empirical construct of our study. Our model predicts the geographical locations of Twitter users across 139 countries. We exclude, however, the countries where Twitter is blocked (i.e., China, North Korea, Russia, Iran, Uzbekistan, Turkmenistan, and Belarus), which account for 0.62% of the users in our sample. Our findings are robust to including these countries in the analysis.

We categorize a corporate fake news story as foreign originated if its rumor source (i.e., the Twitter user who initiates the cascade) is outside the U.S. To put it differently, we define a continuous variable—*Foreign Corporate Fake News (%)*—as the percentage of original tweets spreading fake news initiated by an account in a foreign country. For example, if we have 10 original tweets spreading a fake news story, and five of the tweets are initiated by foreign accounts, the value of *Foreign Corporate Fake News (%)* is 50%. The measure accounts for the fact that fake news can be spread via multiple cascades on Twitter. In robustness tests, we replace *Foreign Corporate Fake News (%)* with *Foreign Corporate Fake News (dummy)*, as binning the data can reduce measurement errors caused by a noisy continuous variable [40]. We define *Foreign Corporate Fake News (dummy)* as an indicator variable equal to one if at least one Twitter account spreading fake news is located in a foreign country, and zero otherwise. We also use corporate non-fake news to construct a corresponding benchmark. We define *Foreign Corporate Non-fake News (dummy)* as an indicator variable equal to one if at least one Twitter account spreading non-fake news is located in a foreign country, and zero otherwise. Similarly, *Foreign Corporate Non-fake News (%)* is the percentage of original tweets spreading non-fake news initiated by an account outside the U.S. We construct the measures at the news level. For much of the analysis, however, we aggregate the measures to the country-year (Section 3.3) and firm-year (Section 3.4) level.

### 3.2. Sampling design and summary statistics

We begin our analysis by reporting descriptive statistics. First, we report data across fake and non-fake corporate news. We identify 541 fake news stories (about 126 unique firms) and 144 non-fake news stories (about 67 unique firms) over the period from January 2012 to June 2021. Within each news category, we tabulate news content and provide statistics about the characteristics of rumor starters. Table 3, Panel A presents the results. Most of the fake news revolves around the firm’s politics, product, or operations. 37.7% of corporate fake news involves political discussions (e.g., support for social movements such as Black Lives Matter, funding of political parties, government contracts, etc.), whereas product-related news (e.g., contaminated products, product discontinuation, product safety, etc.) accounts for 22.6%, and operations-related news (e.g., downsizing, employee policy, etc.) accounts for 16.8% of the fake news. The distribution of topics for non-fake news follows a similar pattern, and the difference across news categories is not statistically significant.

**Table 3:** Characteristics of corporate fake news.

**Panel A.** News content									
	*Fake News*				*Non-fake News*				
	Mean	Stdev	#Obs		Mean	Stdev	#Obs.		t-stats of of
	(1)	(2)	(3)		(4)	(5)	(6)		Diff.
*Data Privacy*	0.046	0.210	25		0.056	0.230	8		-0.01
*Founder/Executive Management*	0.068	0.253	37		0.048	0.216	7		0.02
*Product*	0.226	0.418	122		0.208	0.408	30		0.02
*Politics*	0.377	0.485	204		0.361	0.482	52		0.02
*Religion*	0.061	0.240	33		0.055	0.230	8		0.01
*Operations*	0.168	0.374	91		0.194	0.397	28		-0.03
*Other*	0.054	0.225	29		0.076	0.267	11		-0.02
Total			541				144		
**Panel B.** Twitter data									
	*Fake News*				*Non-fake News*				
	Mean	Stdev	#Obs		Mean	Stdev	#Obs.		t-stats of
	(1)	(2)	(3)		(4)	(5)	(6)		Diff.
*% Foreign Originating Tweets*	37.56%	0.34	294		30.84%	0.32	87		6.72%**
*# Retweets*	482	1,946.15	294		21	84.94	87		461**
*Rumor Starters*:									
*# Followers*	66,672	692,808	152,908		23,762	815,503	64,955		42,910***
*# Followees*	1,686	7,023	152,908		1,780	8,137	64,955		-94***
*Account Age (years)*	5.47	3.38	152,097		6.43	3.60	64,909		-0.96***
*Bot Score*	0.51	0.24	136,539		0.46	0.24	55,605		0.05***
**Panel C.** Distribution of news by industry									
	*# Fake News*	*# Non-fake News*			*% Foreign-originating Fake News Tweets*		*% Foreign-originating Non-fake News Tweets*		
*Fama-French 12 Industry*	(1)	(2)			(3)		(4)		
*Consumer Nondurables*	54	14			35.86%		37.63%		
*Consumer Durables*	14	2			22.74%		0.00%		
*Manufacturing*	16	4			23.81%		21.78%		
*Oil*, *Gas*, *and Coal Extraction and Products*	4	0			0.00%		0.00%		
*Chemicals and Allied Products*	17	3			35.63%		12.50%		
*Business Equipment*	135	26			41.29%		46.82%		
*Telephone and Television Transmission*	57	15			31.79%		27.80%		
*Utilities*	1	1			0.00%		0.00%		
*Wholesale*, *Retail*, *and Some Services*	177	61			30.67%		28.80%		
*Healthcare*, *Medical Equipment*, *and Drugs*	25	3			55.10%		34.49%		
*Finance*	7	3			28.44%		31.10%		
*Other*	34	12			27.54%		31.45%		
**Panel D.** Distribution of news by year									
	*# Fake News*	*# Non-fake News*		*% Foreign-originating Fake News Tweets*				*% Foreign-originating Non-fake News Tweets*	
*Year*	(1)	(2)		(3)				(4)	
*2012*	16	1		39.08%				60.78%	
*2013*	22	3		37.63%				30.92%	
*2014*	26	6		35.45%				30.45%	
*2015*	102	42		39.55%				29.87%	
*2016*	67	10		36.42%				44.14%	
*2017*	52	8		34.43%				37.38%	
*2018*	64	12		36.19%				22.33%	
*2019*	49	19		41.69%				31.87%	
*2020*	91	25		36.53%				27.02%	
*2021*	52	18		37.02%				35.81%	
**Panel E.** Distribution of news by fact-checking organizations									
	*# Fake News*	*# Days to Factcheck*		*# Non-fake News*				*# Days to Factcheck*	
*Snopes*	360	24.09		76				24.86	
*Politifact*	95	9.74		8				15.57	
*Truthorfiction*	83	28.16		60				44.72	
*Factcheck*	3	3.33		0				-	
**Panel F.** Distribution of source news by dissemination platforms									
	*# Fake News*	*# Non-fake News*							
*Social Media*	230	41							
*News Site*	74	44							
*Blog/Forum*	25	6							
*Video Hosting*	14	3							
*NGO*	4	6							
*Other*	8	10							

This table presents an overview of corporate news verified by fact-checking organizations from January 2012 to June 2021. Panel A and Panel B tabulate the news content and the characteristics of news disseminating on Twitter, respectively. Panel C tabulates the distribution of news and percentage of foreign-originating fake news tweets by industry. Panel D tabulates the distribution of news and percentage of foreign-originating fake news tweets over time. Panel E reports the distribution of news and number of days it takes fact-checking organizations to fact-check news. Panel F reports the distribution of news by source.

In Panel B, we identify the individual characteristics of the rumor starters. We use Twitter metadata to extract user profiles. Specifically, we check the number of people who follow the user on Twitter (i.e., followers), the number of people whom the user follows in Twitter (i.e., followees), the age of the user’s account (measured in years), and the percentage of bot accounts. We find that starters of fake news (i.e., Twitter users who initiate the news) have more followers than starters of non-fake news. They are also relatively new accounts. We also use Botometer, a popular public tool, for bot detection on Twitter (see S4 Appendix in S1 File for details). Using this tool, we calculate a *Bot Activity* score between zero and one for each Twitter user. A higher score indicates a higher likelihood that a Twitter account is a bot. We find that bot score is higher for fake news accounts (0.51 versus 0.46), suggesting that fake news spreaders are more likely to exhibit bot-like behavior. We also find that bot-like activity is significantly higher for fake news than for non-fake news, which indicates that a large group of automatically operated accounts may be promoting the fake news (*t* = 36.48, *p*<0.00). Finally, we find that corporate fake news, on average, is retweeted around 480 times, whereas non-fake news is retweeted around 20 times. This finding is consistent with the faster diffusion of fake news on social media, as documented by [10].

In Panels C and D, we report the distribution of news categories across industries and over time. At the industry level, the wholesale, retail, business equipment, and consumer nondurable industries are more likely to be targeted by fake news (columns 1 and 2). Fake news, however, is more likely to be foreign originated in the finance, chemicals, and consumer durables industries (column 3). In temporal trends, the fake news peaks in 2015, after which it gradually starts to decline (Panel D). Panel E reports the distribution of news by fact-checking organizations. Most of the fact-checked news is collected by Snopes (67%). We also calculate the number of days it takes for a fact-checking organization to check a claim. To do this, we manually read and identify the original date of the claim (i.e., the source news). We then subtract the publication date of the fact-checking article from the date when the source news began to disseminate news in the public domain. We find Politifact to be faster than other organizations in fact-checking the claims. We also manually identify the platforms where the source news is disseminated. Panel F reports the results. We find that the fake news is disseminated mostly on social media (e.g., Twitter, Facebook, Youtube). In contrast, non-fake news originates mostly from news sites, consistent with the filtering role that the editorial process plays in traditional media.

### 3.3 Country-level analysis

We next explore the distribution of corporate news at the country level. Our analysis is motivated by the fact that ideologically motivated foreign actors may spread misinformation to attack the reputation of a country’s firms. For example, a recent study finds evidence of social media manipulation campaigns in 70 countries organized by government agencies to shape public attitudes [41]. Several state actors target foreign countries to influence global audiences, amplify hate speech, or harass political figures or journalists, and countries use troll farms in the Middle East and Africa to spread rumors about target countries (see, e.g., https://www.theguardian.com/technology/2020/mar/13/facebook-uncovers-russian-led-troll-network-based-in-west-africa.) The country-level analysis may help shed light on the geographical origin of rumor spreaders (and potential foreign involvement) in capital markets.

Because the number of Twitter users varies across countries, we begin our analysis by normalizing the number of fake (and non-fake) news stories originating from a country by the total number of users in that country in a given year. We use the number of users in the randomly collected geo-tagged tweet dataset as a proxy for the total number of users in that country (as we could not obtain the historical country-level user statistics from Twitter or external data providers). To reduce outlier effects, we include countries with at least 300 tweets in the training data. The top five countries with the highest number of Twitter users are the United States, Indonesia, Brazil, Turkey, and Great Britain. We take the average of the yearly normalized *Foreign Corporate Fake News (%)* at the country-year level to construct our measure at the country level.

**Fig 3:**
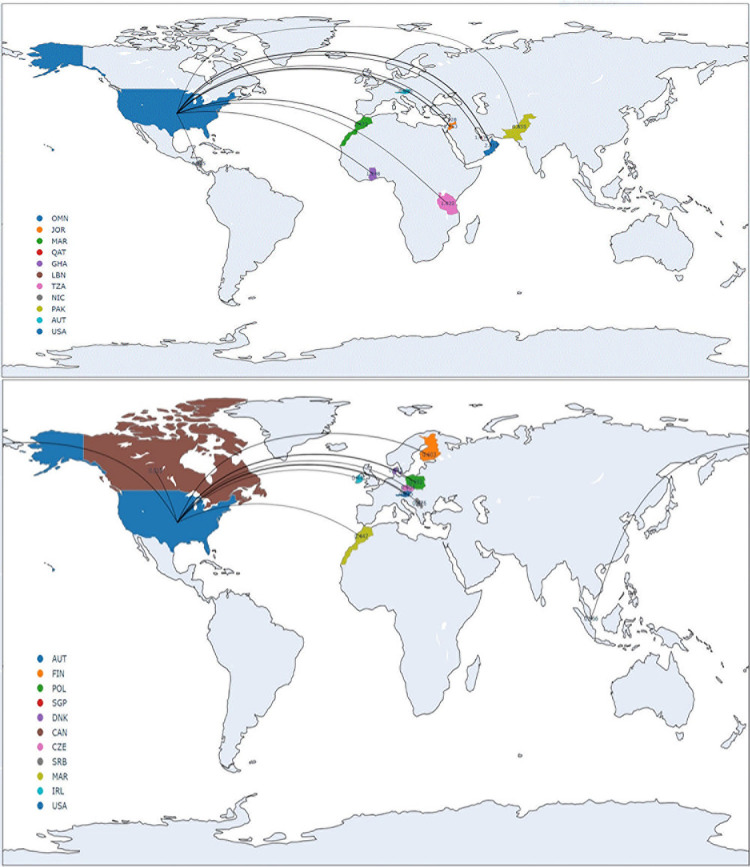
Geographical origins of corporate fake news. This graph plots the geographical location of users spreading corporate news about U.S. public firms. We construct *Foreign Fake News (%)* and *Foreign Non-Fake News (%)* at the country level in two steps. First, we normalize the number of users spreading (fake or non-fake) news on Twitter by the total number of users in each country. We then take the averages of this normalized country-year-level measure to convert it to a country-level measure. In Panel A, we plot the top 10 countries spreading corporate fake news about U.S. public firms. In Panel B, we plot the top 10 countries spreading corporate non-fake news about U.S. public firms. The map in the figure is made with Natural Earth (https://www.naturalearthdata.com/about/terms-of-use/).

Table 4 reports the results. In Panel A, we show that corporate fake news originates primarily from Middle Eastern and African countries. Oman, Jordan, Morocco, Qatar, and Ghana are the top five countries from which most tweets with corporate fake news originate. The probability of each Omani user originating corporate fake news is 2.01%, while the probabilities for Jordanian and Moroccan users are 1.76% and 1.47%, respectively. In contrast, non-fake news originates mostly from Western countries. The probability of each Austrian user originating non-fake news is 0.63%, while the probabilities for Finnish and Polish users are 0.60% and 0.59%, respectively. In Fig 3, we plot the geographical distribution of 10 countries spreading fake (Panel A) and non-fake (Panel B) news about U.S. public firms. In Panel B, to account for the general business interests of users from a specific country, we construct an adjusted measure by subtracting the probability of disseminating non-fake news from that of disseminating fake news. Using this measure, we still find that the foreign fake news originates primarily from the Middle East and Africa (i.e., Oman, Jordan, Qatar, and Morocco), consistent with the anecdotal evidence that foreign actors use troll farms in this region. For example, a Russian-led network of professional trolls (operated by local residents) targeting the U.S. was discovered in Ghana and Nigeria [42]. There is also evidence of Lebanese, Nicaraguan, and Moroccan governments running disinformation campaigns for political motives (see, e.g., https://www.newarab.com/analysis/disinformation-and-electronic-armies-lebanons-elections for Lebanon, see https://www.bbc.com/news/world-latin-america-59129894 for Nicaragua, and see https://www.accessnow.org/how-pro-government-media-in-morocco-use-fake-news-to-target-and-silence-rif-activists/ for Morocco).

**Table 4:** Geographical distribution of corporate fake news.

**Panel A.** Top 10 countries			
Country	*% Fake News*	Country	*% Non-fake News*
*Oman*	2.01	*Austria*	0.63
*Jordan*	1.76	*Finland*	0.60
*Morocco*	1.47	*Poland*	0.59
*Qatar*	1.43	*Singapore*	0.57
*Ghana*	1.25	*Denmark*	0.54
*Lebanon*	1.23	*Canada*	0.52
*Tanzania*	1.02	*Czechia*	0.50
*Nicaragua*	0.93	*Serbia*	0.48
*Pakistan*	0.86	*Morocco*	0.45
*Austria*	0.85	*Ireland*	0.44
**Panel B.** Top 10 countries (adjusted)			
Country	*% Fake News—% Non-fake News*		
*Oman*		1.81	
*Jordan*		1.39	
*Qatar*		1.08	
*Morocco*		1.03	
*Lebanon*		0.96	
*Ghana*		0.91	
*Tanzania*		0.65	
*Nicaragua*		0.63	
*Pakistan*		0.60	
*Egypt*		0.55	

This table reports the top 10 countries from which corporate fake and non-fake news originate. For the analysis, we construct a country-level measure in two steps. First, we normalize the number of users spreading (fake or non-fake) news on Twitter by the total number of users in each country. We then take the yearly averages of this normalized country-year-level measure to convert it to a country-level measure. Panel A reports the top 10 countries spreading corporate fake and non-fake news. Panel B reports the same ranking using an adjusted measure taking the difference between *% Fake News* and *% Non-fake News*.

Next, we employ the KL divergence metric to compare the country distribution of the percentage of Twitter accounts spreading fake and non-fake news. KL divergence is a measure of how one probability distribution differs from a second distribution. Intuitively, we examine how far away the geographical distribution of the percentage of accounts spreading fake news is from the geographical distribution of the accounts spreading non-fake news. If the two distributions match perfectly, KL divergence is zero; otherwise, it can take values between zero and infinity. We find that the KL divergence is 0.62 and statistically significant (*SE* = 0.13, 95% CI[0.37,0.88]), which suggests that the geographical distribution of fake news is different from that of non-fake news. We also compare the concentration of geographic locations using a median relative polarization index (MRP). A positive MRP implies a more uneven distribution of news across countries relative to a benchmark. We take the distribution of locations spreading non-fake news as our benchmark and compute the MRP. We find that the MRP is relatively higher within countries spreading fake news (*MRP* = 0.33, *SE* = 0.11, 95% CI[0.12,0.54]), suggesting that the distribution of countries spreading fake is more concentrated than that of countries spreading non-fake news.

Finally, we examine if the probability of foreign fake news is more pronounced during periods of high geopolitical risks. To do this, we plot a time series of the news originating from foreign accounts and geopolitical risks. We use two proxies for geopolitical risks. First, we employ a news-based measure of the *Geopolitical Risk Index (GPR)* developed by [43]. The authors of [43] (on page 1195) define geopolitical risks “as the threat, realization, and escalation of adverse events associated with wars, terrorism, and any tensions among states and political actors that affect the peaceful course of international relations.” They construct an index at the country-year level by counting the share of articles mentioning adverse geopolitical events in leading newspapers in the U.S. A higher level of *GPR* corresponds to escalated geopolitical tensions facing the U.S. Second, we use the Global Database on Event, Location, and Tone (GDELT) to construct an *Interstate Conflict Index*. GDELT is the most widely used database for studying international relations and conflicts. It contains more than 200 million geolocated events compiled from international news sources [44]. The sources include AfricaNews, Agence France Presse, Associated Press, BBC Monitoring, United Press International, The Washington Post, The New York Times, and Google News. After machine coding the relevant information in the text of a news story (e.g., related countries, type of event, intensity of conflict or cooperation) into events, GDELT merges all duplicate events into a single event record. GDELT then provides the Goldstein scale [45], measuring the impact of each event from -10 (most conflictual) to +10 (most cooperative). We calculate the annual average Goldstein scale between the U.S. and other countries and reverse the scale to make it an increasing function of the conflicts that the U.S. faces.

**Fig 4:**
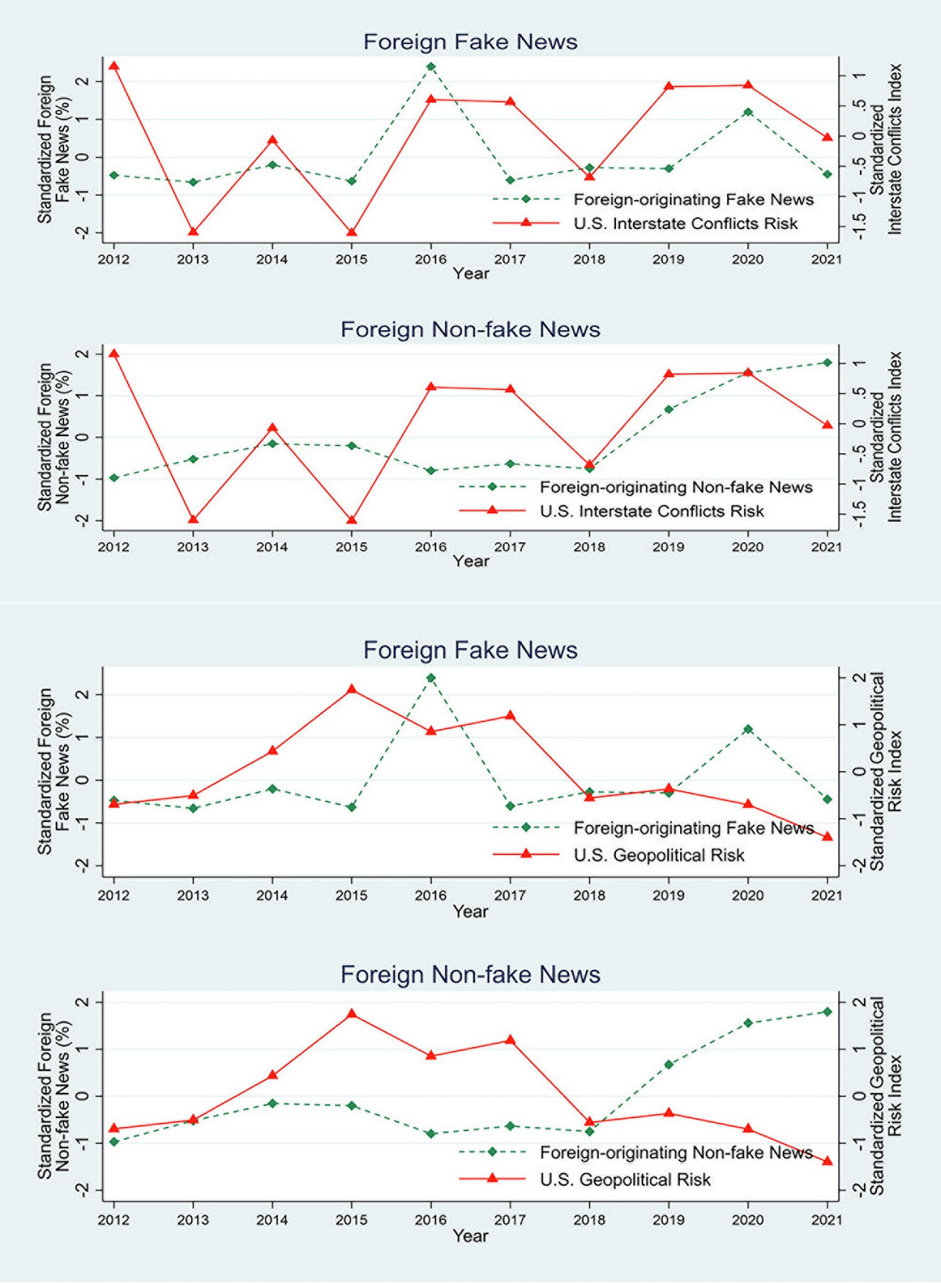
Foreign-originating fake news and geopolitical tensions. This figure plots the time series of corporate news originating from foreign countries and geopolitical tensions. *Foreign Fake News (%)* is the percentage of original tweets spreading corporate fake news initiated by a foreign (non-U.S.) Twitter account. We reconstruct this measure by aggregating all the corporate news at the yearly level. Panel A plots the relation between *Foreign (Non-fake) Fake News (%)* and *Interstate Conflict Risk*. *Interstate Conflict Risk* is an index measuring interstate conflicts (based on the Goldstein scale) using daily reported events in the global news media. Panel B plots the relation between *Foreign Fake (Non-fake) News (%)* and *Geopolitical Risk Index*. *Geopolitical Risk Index* is the share of articles mentioning adverse geopolitical events in leading newspapers in the U.S. For ease of interpretation, we standardize the indices and news measures.

We use these two indices—*GPR* and the *Interstate Conflict Index*—to explore the relation between the news originating from foreign accounts and geopolitical tensions. We calculate the annual average of *Foreign Corporate Fake News (%)* (at the U.S. level) to capture the prevalence of foreign accounts targeting U.S. firms. For ease of interpretation, we standardize *Foreign Corporate Fake News (%)* and the indices. We plot the temporal evolution of the variables in Fig 4.

Panel A shows the close comovement of *Foreign Corporate Fake News (%)* and *Interstate Conflict Index*. There is a positive correlation (0.40) between the proportion of fake news from foreign accounts and interstate conflict risk. The correlation, however, is weaker (0.17) for *Foreign Corporate Non-fake News (%)*. We observe a similar pattern in Panel B using *GPR Index*. The correlation between *GPR* and *Foreign Corporate Fake News (%)* is positive (0.09), but that between *GPR* and *Foreign Corporate Non-fake News (%)* is negative (-0.48). Overall, the increased fake news originating from foreign countries during periods of high geopolitical tensions suggests a link between foreign accounts and state actors. However, given our data limitations and descriptive research design, we interpret our evidence as merely suggestive.

### 3.4 Firm-level analysis

It is an empirical question what kind of firms are more likely to be targeted by fake news.

At the country level, we show a comovement between heightened geopolitical risks and foreign-originating fake news. At the firm level, we predict that firms in strategic industries (i.e., the telecommunication, pharmaceutical, semiconductor, military, and computer industries) and firms that are leaders in their industries are more likely to be targeted by foreign actors, given the recent proliferation of foreign-originated cyberattacks that have damaged the reputations of targeted firms in strategic industries (see, e.g., https://nyti.ms/3jsdGSR, https://bit.ly/3twMIho). Similarly, foreign actors can strategically disseminate fake news on social media to tarnish the reputations of strategically important firms, as reputation is often considered a firm’s most important intangible asset [46]. A significant body of research shows the effect of reputation on asset prices [47, 48], firm sale [49], risk and financial policy [50], investor preferences [51], and consumer behavior [52, 53]. Ideologically motivated actors may use Twitter to strategically target important industries, as reiterated news conveyed via multiple channels can reach a wide audience of individual and institutional investors [20].

To empirically test this prediction, we construct *Foreign Fake News (%)* by identifying the proportion of fake news originating from foreign accounts for a specific firm in a given year. For example, if 100 accounts initiate fake news about a firm in a given year, and 40 of these accounts are from a foreign country, *Foreign Fake News (%)* is 40%. We employ both univariate and regression analyses to examine the characteristics of firms targeted by fake news. Table 5 Panel A reports univariate results. Our firm-level analysis does not include 22 (11) private firms that are subject to fake (non-fake) news because we do not have financial data for this subset of firms. In the sample, we have 59 unique public firms (465 firm-years) targeted by fake news and 17 unique public firms (145 firm-years) subject to non-fake news. The firms targeted by fake news are significantly more profitable and have greater foreign sales. Target firms have higher pooled average *Return on Assets* (ROA) (0.12 versus 0.08) and lower *Book-to-Market* ratio (0.30 versus 0.50). Industry competition (as measured by *TNIC HHI*) also increases the probability of a firm being targeted by fake news. The *TNIC HHI* of target firms (0.35) is higher than that of firms with non-fake news (0.24). Firms in strategic industries (i.e., pharmaceutical, semiconductor, computer, defense, and telecommunication) are more likely to be targeted by fake news (0.22 versus 0.12). More importantly, the fake news is more likely to originate from foreign accounts. 14.21% of fake news is initiated by a non-US Twitter account versus 6.37% of non-fake news (see S5 Appendix in S1 File for the variable definitions).

**Table 5:** Determinants of being targeted by foreign-originating fake news.

**Panel A.** Characteristics of firms with fake and non-fake news on Twitter						
	*Fake News (N = 465)*		*Non-fake News (N = 145)*		*Diff*. *(Fake News–Non-fake News)*	
	Mean	Std. Dev.	Mean	Std. Dev.	Diff. in Means	t-stat of Diff.
	(1)	(2)	(3)	(4)	(5)	(6)
*% Foreign-originating Tweets*	0.14	0.25	0.06	0.18	0.08*** 0.000	0.00
*Total Assets (ln)*	10.27	1.70	10.54	2.17	-0.26	0.13
*Book-to-Market*	0.30	0.27	0.50	0.30	-0.21***	0.00
*Loss*	0.09	0.28	0.09	0.29	-0.00	0.89
*Dividend Dummy*	0.80	0.40	0.85	0.36	-0.05	0.18
*Return on Assets*	0.12	0.10	0.08	0.15	0.04***	0.00
*Leverage*	0.31	0.18	0.30	0.15	0.02	0.35
*Sales Growth*	0.12	0.49	0.07	0.17	0.06	0.17
*Foreign Sales*	0.40	0.49	0.23	0.42	0.18***	0.00
*Product Similarity*	4.00	9.63	6.45	12.76	-2.46**	0.01
*TNIC HHI*	0.35	0.28	0.24	0.22	0.11***	0.00
*Institutional Ownership*	0.57	0.33	0.57	0.35	0.00	0.94
*Return Volatility*	0.02	0.01	0.02	0.01	-0.00	0.50
*Strategic Industry*	0.22	0.41	0.12	0.32	0.10***	0.00
*Industry Leader*	0.31	0.47	0.32	0.47	-0.00	0.94
**Panel B.** Foreign fake and non-fake news						
	*Dependent variable*:					
	*Foreign Fake News (%)*	*Foreign Non-fake News (%)*	*Chi-square (χ* ^*2*^ *) of Differences*	*Foreign Fake News (%)*	*Foreign Non-fake News (%)*	*Chi-square (χ* ^*2*^ *) of Differences*
Independent Variables	(1)	(2)	(3)	(4)	(5)	(6)
*Total Assets (Ln)*	0.003***	0.002***	0.02***	0.003***	0.002***	0.06*
	(4.69)	(3.90)		(4.15)	(3.57)	
*Book-to-Market*	-0.002***	-0.001***	0.02**	-0.003***	-0.001***	0.04**
	(-3.26)	(-2.60)		(-3.82)	(-3.53)	
*Loss*	-0.000	0.000	0.30	0.000	0.000	0.98
	(-0.37)	(0.62)		(0.97)	(0.93)	
*Dividend Dummy*	0.000	-0.001	0.21	-0.001	-0.002*	0.22
	(0.38)	(-0.68)		(-0.81)	(-1.76)	
*Return on Assets*	0.000	-0.001	0.36	0.002	0.000	0.29
	(0.18)	(-0.97)		(1.20)	(0.42)	
*Leverage*	-0.004	-0.003	0.35	-0.000	-0.003	0.32
	(-1.62)	(-1.37)		(-0.13)	(-1.55)	
*Sales Growth*	0.000	-0.000	0.49	0.001	-0.000	0.16
	(0.61)	(-0.18)		(1.28)	(-0.34)	
*Foreign Sales*	0.002**	0.000	0.06*	0.002*	-0.000	0.03**
	(2.09)	(0.33)		(1.99)	(-0.68)	
*Product Similarity*	0.000	-0.000	0.26	-0.000	-0.000*	0.82
	(0.76)	(-0.29)		(-1.14)	(-1.73)	
*TNIC HHI*	0.005**	0.006**	0.99	0.007**	0.005**	0.49
	(1.98)	(2.21)		(2.49)	(2.36)	
*Institutional Ownership*	-0.010***	-0.006***	0.02**	-0.008***	-0.005***	0.05**
	(-4.74)	(-3.89)		(-4.09)	(-3.69)	
*Return Volatility*	0.027**	0.010	0.08*	0.022**	0.016**	0.55
	(2.26)	(1.37)		(1.98)	(2.41)	
*Strategic Industry*				0.004**	0.002*	0.10*
				(2.32)	(1.94)	
*Industry Leader*				0.021**	0.016***	0.44
				(2.40)	(2.77)	
*Industry Fixed Effects*	Yes	Yes		No	No	
*Year Fixed Effects*	Yes	Yes		Yes	Yes	
*Firm Clustering*	Yes	Yes		Yes	Yes	
# Observations	26,603	26,534		26,603	26,534	
Adjusted-R^2^	0.088	0.059		0.031	0.021	

This table shows both univariate and regression analyses of the determinants of being targeted by fake news. Panel A reports the characteristics of firms targeted at least once by fake news in columns 1 and 2 and firms subject to non-fake news in columns 3 and 4. Columns 5 and 6 report the difference in means, with the corresponding t-statistics. Panel B reports results from the linear regression model to examine the likelihood of a firm being targeted by fake news originating from foreign Twitter accounts. The dependent variable is *Foreign Fake News (%)* in columns 1 and 4, and *Foreign Non-fake News (%)* in columns 2 and 5. In columns 3 and 6, we compare the coefficient estimates across the models. We estimate the model with industry and year fixed effects in columns 1 and 2. We exclude industry fixed effects in columns 4 and 5 when estimating the model with *Strategic Industry* and *Industry Leader*. Standard errors are clustered at the firm level, and t-statistics are in parentheses. Intercepts are included for estimation but not tabulated. All variables are defined in S5 Appendix in S1 File. ***, **, and * indicate two-tailed t-statistics with statistical significance at the 1%, 5%, and 10% level, respectively.

In Table 5 Panel B, we estimate the following model to examine the likelihood of a firm being targeted by fake news originating from foreign Twitter accounts:

$$Foreign\;Fake(Non‐fake)\;News_{it}=\alpha_{it}+\beta_{1}Total\;Assets_{it}+\beta_{2}Book‐to‐market_{it}+\beta_{3}Loss_{it}+\beta_{4}Dividend\;Dummy_{it}+\beta_{5}Return\;on\;Assets_{it}+\beta_{6}Leverage_{it}+\beta_{7}Sales\;Growth_{it}+\beta_{8}Foreign\;Dummy_{it}+\beta_{9}TNIC\;HHI_{it}+\beta_{10}Product\;Similarity_{it}+\beta_{11}Return\;Volatility_{it}+\beta_{12}Institutional\;Ownership_{it}+\beta_{13}Industry\;Leader_{it}+\beta_{14}Strategic\;Industry_{it}+\alpha_{t}+\alpha_{s}+\varepsilon_{it}$$ (1)

where *Foreign Fake (Non-fake) News (%)* is the percentage of fake (non-fake) news spread by foreign accounts for a specific firm a given year. *i*, *t*, and *s* denote the firm, year, and industry subscripts, respectively. We use year and industry fixed effects to control for temporal trends and time-invariant industry characteristics. Because we are interested in the across-industry variation, we exclude industry fixed effects when estimating the model with *Strategic Industry* and *Industry Leader*. Standard errors are clustered at the firm level to avoid underestimation [54]. A firm can be exposed to both fake and non-fake news in a given year. Therefore, in the empirical analysis, we separately analyze firm-years where there is fake or non-fake news (and compare them with firm-years without fact-checked news).

In the model, we include a vector of firm-level characteristics, including size (*Total Assets*), profitability (*Return on Assets)*, leverage (*Leverage*), book-to-market ratio (*Book-to-Market*), dividend (*Dividend Dummy*), and a loss dummy (*Loss*). We do not have a clear directional prediction for these variables. To capture a firm’s visibility in foreign markets, we also control for *Foreign Sales*, defined as a dummy variable equal to one if at least 10% of a firm’s sales are to foreign (non-U.S.) markets. In addition, we control for *TNIC HHI* and *Product Similarity* to capture market competition. *TNIC HHI* is calculated as the sum of the squared market shares of all firms operating in the same industry, using the time-varying Text-based Network Industry Classification (TNIC) developed by [55]. *Product Similarity* is a firm-level measure based on product descriptions from 10-K filings [55]. We include these measures because peer firms can spread disinformation about their rivals, especially in competitive markets [25]. For example, the authors of [25] document negative peer disclosure (NPD) as an emerging corporate strategy firms use to publicize adverse news about their industry peers on social media. Moreover, NPD propensity increases with product market rivalry, as highly competitive industries provide greater incentives to spread negative news.

We employ *Institutional Ownership* and *Return Volatility* to control for the relation between the information environment and the fake news. *Institutional Ownership* represents the percentage of a firm’s stock held by institutional investors. *Return Volatility* captures uncertainty regarding a firm’s underlying fundamentals, which we measure as the standard deviation of a firm’s daily stock returns during a fiscal year. A poor information environment can create incentives for rumormongers to spread fake news, as investors are more likely to be influenced by news when access to alternative information sources is limited. We use these variables to control for the relation between a firm’s information environment and the likelihood of being targeted by fake news.

Table 5, Panel B summarizes the results. In column 1, we use *Foreign Fake News (%)* as the dependent variable in the baseline model. In column 2, we estimate a benchmark model using *Foreign Non-fake News (%)* for comparison. We find that larger firms with foreign sales and growth opportunities are more likely to be targeted by foreign fake news. Column 3 compares the coefficients across the baseline and benchmark models. The coefficient estimates of target firms are also significantly different from those of non-target firms. Second, we find that firms operating in less competitive markets (i.e., industries with higher *TNIC HHI*) are more likely to be targeted by foreign fake news. The coefficient estimate on *TNIC HHI*, however, is not statistically different from that on non-fake news (*χ*^*2*^ = 0.00, *p*<0.99).

Next, we examine whether firms with an uncertain information environment (i.e., higher return volatility) and a less sophisticated investor base (i.e., high retail ownership) are more prone to foreign-originating fake news. Information frictions may slow the price-discovery process and can cause prices to deviate from intrinsic values for prolonged periods [56, 57], which increases the influence of rumormongers on stock prices. Theoretical literature suggests that rumors create profit opportunities for rumormongers [58], and recent work shows that corporate fake news can affect stock prices [11, 59, 60]. Consistent with our conjecture, we find the incidence of fake news to be higher for firms that have a less robust information environment. The likelihood of being targeted by a foreign source is higher for firms with lower institutional ownership (-0.010 versus -0.006, *χ*^*2*^ = 5.63, *p*<0.02) and higher return volatility (0.027 versus 0.010, *χ*^*2*^ = 3.07, *p*<0.08).

In columns 4 and 5, we examine whether fake news from foreign accounts is concentrated in strategically important firms. To test this, we use two variables. First, we define *Industry Leader* as an indicator equal to one if a firm is the largest member of its industry in terms of revenue, and zero otherwise. Second, we define *Strategic Industry* as an indicator equal to one if a firm is in the computer, telecommunication, pharmaceutical, semiconductor, or defense industry, and zero otherwise. We predict and find that firms that are leaders in their industries, as well as firms operating in strategic industries, are more likely be targeted by fake news originating from foreign countries. Being a member of a strategic industry increases the proportion of fake news from foreign countries by 2.9% (0.004/0.137). The coefficient estimate on *Strategic Industry* for target firms (0.004) is statistically different from the coefficient estimate for non-target firms (0.002) with *χ*^*2*^ = 2.63 and *p*<0.10. Industry leaders are also more likely to be targeted by foreign accounts (0.021 versus 0.016). However, the difference of coefficient estimates is not statistically significant between firms subject to fake and non-fake news (*χ*^*2*^ = 0,58 and *p*<0.45).

Overall, the firm-level analysis complements our country-level findings by showing that strategic industries have a higher probability of being targeted by fake news originating from foreign countries. That said, as discussed in the Introduction, we cannot cleanly attribute our findings to a foreign state actor or an intentional disinformation operation. Other potential reasons for disseminating negative fake news include the financial incentives of investors holding short positions or participating in other schemes that would benefit from a negative market reaction. In addition, inter-firm competition incentives may induce competitors to spread false news about their rivals. Finally, social media algorithms may unintentionally amplify certain content, including misinformation, based on factors like user engagement or click-through rates. While these are all plausible possibilities, they do not line up well with our country- and firm-level findings (i.e., the positive correlation between foreign-originated fake news and heightened geopolitical risks, as well as the targeting of strategic industries). We acknowledge, however, that we cannot fully rule out these alternative explanations, given the aforementioned limitations and the lack of granular data.

3.5 Additional analysis

In this section, we conduct three additional tests. First, we exclude political corporate fake news from our sample as politics is the most common topic found in corporate fake news (37.7%). Table 6, Panel A summarizes the results. Our analysis shows that the results hold in the sample of fake and non-fake corporate news (except that some differences in the coefficient estimates are statistically insignificant).

**Table 6:** Additional analysis.

**Panel A.** Excluding political corporate fake news						
	*Dependent variable*:					
	*Foreign Fake News (%)*	*Foreign Non-fake News (%)*	*Chi-square (χ* ^*2*^ *) of Differences*	*Foreign Fake News (%)*	*Foreign Non-fake News (%)*	*Chi-square (χ* ^*2*^ *) of Differences*
Independent Variables	(1)	(2)	(3)	(4)	(5)	(6)
*Total assets (Ln)*	0.002***	0.002***	0.04**	0.002***	0.001***	0.03**
	(4.19)	(3.23)		(3.78)	(2.93)	
*Book-to-Market*	-0.001**	-0.000**	0.15	-0.002***	-0.001***	0.02**
	(-2.41)	(-2.07)		(-3.42)	(-3.14)	
*Loss*	-0.000	0.000	0.15	0.000	0.000	0.98
	(-0.80)	(0.59)		(0.75)	(0.74)	
*Dividend Dummy*	0.000	-0.000	0.25	-0.001	-0.001*	0.64
	(0.20)	(-0.67)		(-1.08)	(-1.75)	
*Return on Assets*	0.001	0.000	0.68	0.001	0.001	0.49
	(0.42)	(0.03)		(1.11)	(0.75)	
*Leverage*	-0.003*	-0.002	0.50	-0.001	-0.002	0.99
	(-1.79)	(-1.36)		(-0.43)	(-1.38)	
*Sales Growth*	0.000	0.000	0.89	0.000	0.000	0.36
	(0.30)	(0.57)		(1.22)	(0.64)	
*Foreign Sales*	0.002**	0.000	0.07*	0.002**	0.000	0.19
	(2.05)	(0.79)		(1.97)	(0.12)	
*Product Similarity*	0.000	-0.000	0.15 030	0.000	-0.000	0.27 030
	(0.62)	(-0.77)		(0.30)	(-1.46)	
*TNIC HHI*	0.005**	0.003*	0.22	0.006**	0.003**	0.03**
	(2.10)	(1.70)		(2.43)	(2.30)	
*Institutional Ownership*	-0.006***	-0.004***	0.08*	-0.005***	-0.003***	0.11
	(-3.81)	(-3.21)		(-3.73)	(-3.06)	
*Return Volatility*	0.021*	0.008	0.12	0.015*	0.009*	0.30
	(2.01)	(1.09)		(1.86)	(1.76)	
*Strategic Industry*				0.002*	0.001	0.21
				(1.93)	(1.35)	
*Industry Leader*				0.014**	0.011**	0.30
				(2.08)	(2.27)	
*Industry Fixed Effects*	Yes	Yes		No	No	
*Year Fixed Effects*	Yes	Yes		Yes	Yes	
*Firm Clustering*	Yes	Yes		Yes	Yes	
# Observations	26,536	26,496		26,536	26,496	
Adjusted-R^2^	0.082	0.050		0.022	0.014	
**Panel B.** Foreign fake vs. true news (Dummy)						
	*Dependent variable*:					
	*Foreign Fake _News (Dummy)*	*Foreign Non-fake _News (Dummy)*	*Chi-square (χ* ^*2*^ *) of Differences*	*Foreign Fake _News (Dummy)*	*Foreign Non-fake News (Dummy)*	*Chi-square (χ* ^*2*^ *) of Differences*
Independent Variables	(1)	(2)	(3)	(4)	(5)	(6)
*Total Assets (Ln)*	0.008***	0.005***	0.00***	0.006***	0.004***	0.01**
	(5.01)	(4.33)		(4.42)	(3.81)	
*Book-to-Market*	-0.004***	-0.002***	0.00***	-0.006***	-0.003***	0.01***
	(-3.81)	(-2.99)		(-4.40)	(-3.40)	
*Loss*	0.000	0.000	0.92	0.001	0.001	0.50
	(0.35)	(0.38)		(1.30)	(0.87)	
*Dividend Dummy*	-0.001	-0.001	0.76	-0.003	-0.003*	0.83
	(-0.23)	(-0.65)		(-1.39)	(-1.69)	
*Return on Assets*	-0.001	-0.003	0.49	0.004	0.001	0.24
	(-0.45)	(-1.50)		(1.05)	(0.20)	
*Leverage*	-0.011*	-0.006	0.21	-0.003	-0.004	0.71
	(-1.78)	(-1.59)		(-0.45)	(-1.29)	
*Sales Growth*	0.001	-0.000	0.35	0.001	-0.000	0.16
	(0.86)	(0.14)		(1.32)	(-0.27)	
*Foreign Sales*	0.003*	0.002	0.27	0.002	-0.000	0.13
	(1.79)	(1.15)		(1.35)	(-0.25)	
*Product Similarity*	0.000	0.000	0.23	-0.000	-0.000**	0.85
	(1.05)	(0.01)		(-1.64)	(-2.28)	
*TNIC HHI*	0.014***	0.010**	0.35	0.015***	0.009**	0.13
	(2.61)	(2.13)		(3.02)	(2.33)	
*Institutional Ownership*	-0.024***	-0.014***	0.01**	-0.018***	-0.011***	0.03**
	(-5.10)	(-4.31)		(-4.53)	(-3.82)	
*Return Volatility*	0.048*	0.014	0.08*	0.043*	0.025*	0.34
	(1.89)	(0.94)		(1.88)	(1.91)	
*Strategic Industry*				0.008**	0.005**	0.21
				(2.33)	(2.08)	
*Industry Leader*				0.058***	0.043***	0.28
				(2.95)	(2.78)	
*Industry Fixed Effects*	Yes	Yes		No	No	
*Year Fixed Effects*	Yes	Yes		Yes	Yes	
*Firm Clustering*	Yes	Yes		Yes	Yes	
# Observations	26,603	26,534		26,603	26,534	
Adjusted-R^2^	0.102	0.109		0.045	0.033	

This table shows additional analysis of the determinants of being targeted by fake news. Panel A reports results from the linear regression model to examine the likelihood of a firm being targeted by fake news originating from foreign Twitter accounts. The dependent variable is *Foreign Fake News (%)* in columns 1 and 4, and *Foreign Non-fake News (%)* in columns 2 and 5. *Foreign Fake News (%)* excludes politically motivated corporate news. In columns 3 and 6, we compare the coefficient estimates across the models. We estimate the model with industry and year fixed effects in columns 1 and 2. We exclude industry fixed effects in columns 4 and 5 when estimating the model with *Strategic Industry* and *Industry Leader*. Panel B replicates the regression in Panel A with *Foreign Fake (Non-fake) News (Dummy)* as the dependent variable. *Foreign Fake (Non-fake) News (Dummy)* is an indicator variable equal to one if at least one original tweet spreading corporate fake (non-fake) news is initiated by a foreign (non-U.S.) Twitter account, and zero otherwise. Standard errors are clustered at the firm level, and t-statistics are in parentheses. Intercepts are included for estimation but not tabulated. All variables are defined in S5 Appendix in S1 File. ***, **, and * indicate two-tailed t-statistics with statistical significance at the 1%, 5%, and 10% level, respectively.

Second, in the analysis above, we use *Foreign Fake News (%)* as a continuous measure to capture the prevalence of foreign-originated fake news. Alternatively, we create an indicator variable *Foreign Fake News (dummy)* that is equal to one if a firm is targeted by foreign-originated fake news, and zero otherwise. Unlike the continuous measure, *Foreign Fake News (dummy)* takes a value of one if at least one initiator of fake news is a foreign account. Panel B summarizes the results. The findings are robust to the alternative use of this indicator variable.

Third, our inferences are robust to alternative clustering of standard errors, such as two-way clustering at the firm and year level (untabulated).

## 4. Discussion

This paper exploits a machine-learning approach to infer the geographical distribution of fake news spreaders on Twitter. We find that corporate fake news is more likely to originate from foreign countries, is more pronounced during periods of high geopolitical tension, and is more likely to target strategic industries and firms operating in uncertain information environments.

Our findings have both policy and practical implications. First, they will be of interest to policymakers, as they provide initial evidence of foreign-originating misinformation in capital markets. While we cannot attribute misinformation to a foreign state actor (or an intentional disinformation operation), we provide preliminary evidence that foreign influence operations in the political sphere can carry over to the economic domain. The findings may encourage policymakers to establish fact-checking organizations dedicated to financial information. Or, adopting a proactive approach, they can implement (or develop) advanced AI tools to detect and track misinformation campaigns in real time to combat online falsehood [61]. Educating investors about identifying red flags for misleading information can also help investors make informed decisions.

Second, our findings show the importance of a holistic approach to information risk. In today’s world, companies can be political targets. In this new environment, executives must not focus narrowly on financial or accounting information but must also pay attention to broader information risks (e.g., disinformation campaigns on social media). Geopolitical risks can further incentivize foreign actors to seed fake news about U.S. firms. Our work suggests that executives should plan for disinformation campaigns, be ready to respond to incidents online, and plan for these events in the new geopolitical and information environment. To do this, companies may leverage new technologies to monitor, detect, and respond to misinformation campaigns, e.g., by building internal fact-checking teams, partnering with independent fact-checking organizations, or using generative AI tools. They can actively engage in ‘social media listening’ to monitor real-time online conversations about the firm and gather insights about their brands, industry, or products. In doing so, companies can communicate more accurate information (and debunk false claims) during periods of high misinformation risk.

Our findings provide initial suggestive evidence on the potential involvement of foreign actors in the economic domain. Future work can explore the benefit the rumormongers derive from spreading negative fake news and the ultimate impact of negative fake news on stock market behavior. It would be also interesting to examine the optimal response of firms to the fake news. Finally, investigating the cross-platform spread of misinformation and the use of new technologies could provide a broader perspective. Recent advances in technology (e.g., generative AI) may allow for the creation of highly realistic but entirely fabricated video or audio content. These deepfakes can be used to spread misinformation without relying on traditional text-based methods. We leave these and other considerations for future research.

## Supporting information

S1 File Supporting information files containing S1-S5 Appendices. (PDF)
