# Supplementary material for: The geography of corporate fake news
Source: PLoS One. 2024 Apr 17;19(4):e0301364. doi: 10.1371/journal.pone.0301364 (PMC11023451; doi:10.1371/journal.pone.0301364)
Supplement: S1 File — (PDF) [file pone.0301364.s001.pdf]

Supplementary Appendix

for

The Geography of Corporate Fake News

S1 Appendix: Location-prediction model

S2 Appendix: Conversion of fact-checking categories to fake and non-fake news

S3 Appendix: Final labeling rules for identifying firm-related news

S4 Appendix: Bot activity check

S5 Appendix: Variable definitions

Supplementary Appendix 1
Location-prediction model

In this appendix, we provide technical details about the location-prediction model. We outline the data collection, data pre-processing, and model training step by step.

Step 1: Collecting training data

We collect a global sample of geo-tagged tweets because the geo-tagged locations (based on GPS coordinates of mobile devices) are reliable and difficult to manipulate. We use locations in geo-tagged tweets as ground-truth data and use them to train our model. We obtain the training data from two sources: (i) the authors of [1], who collect a global sample of 5,053,103 geo-tagged tweets in real time in 2014 and 2015, and (ii) Brandwatch, from which we randomly collect tweets over the sample period 2014-2019. The authors of [1] collect the geo-tagged tweets dataset for a location-prediction task and share only the tweet IDs, as Twitter does not allow the sharing of individual tweets (https://figshare.com/articles/dataset/Tweet_geolocation_5m/_3168529). We reconstruct the dataset with tweet IDs using Twitter Academic API, from which we were able to retrieve 2,970,736 tweets (58.8% of the global sample). The remaining tweets are no longer available, either because users deleted these messages, or the user accounts were suspended. To extend the sample period of training data, we randomly collect a global sample of 1,275,110 geo-tagged tweets from Brandwatch, a Twitter data partner that offers access to historical tweets. In doing so, we extend our sample through 2019. We keep only the native Twitter posts (where the geographical data are based on the GPS location of the device) and remove tweets from Instagram crossposts (where the user can choose a place to attach to the tweet in Instagram, which may lead to incorrect locations [2]). After we remove the Instagram tweets, the Brandwatch sample results in a total of 956,827 tweets. In the end, our training data consist of a global sample of 3,927,563 geotagged tweets over the period 2014-2019. We cannot extend the training data to post-2019 as Twitter removed the ability to tag precise locations in 2019 (https://twitter.com/TwitterSupport/status/1141039841993355264).

We pose the prediction task as a multi-label classification of geolocations. Our training data have 3,315 city locations with geocoordinates obtained from the code repository of [3] (https://github.com/Erechtheus/geolocation). The geotagged tweets are mapped to 3,315 cities using the Haversine distance. We keep 2,187 cities that are geotagged by a minimum of 100

tweets to minimize generalization error for rarely mentioned cities. The final training data include 149 countries and 2,187 cities.

Step 2: Pre-processing data

We use the following Twitter features to predict users' geolocations – tweet text, tweet language, user-declared location, user description, and user name. These are the commonly used features in the prior literature [3,4]. Some other features used in the literature (e.g., time zone, UTC, user language) were not accessible via Twitter API at the time of our study. We also do not use URL links and tweet source, as they do not meaningfully contribute to the location prediction [3].

We begin cleaning the text data by (i) removing links, user names, punctuation, and extra spaces, (ii) separating emoticons (using Python emoji package 2.2.0 on https://pypi.org/project/emoji/), and (iii) making all text lower case. Then, we concatenate the features as follows, inserting special tokens in front of each text field. A [BLANK] token is inserted if the specific text field is blank.

'[TEXT] <cleaned_text> [LANG] <tweet_lang> [LOC] <cleaned_user_declared_location> [DESC] <cleaned_user_description> [NAME] <cleaned_user_name>'

Tokenization is the process of breaking up text up into individual tokens. Tweets, however, are more difficult to tokenize than formal text, and tokenization might be challenging for some languages. While some tokenizers may be suitable for most languages (e.g., English and French), they cannot readily be applied to other languages without clear word boundaries (e.g., Japanese). To overcome this challenge, we use BertTokenizerFast (https://huggingface.co/bert-base-multilingual-uncased), which is common in the training of multilingual BERT models. As our Twitter corpus is multilingual and very different from the pre-trained language models, we train the data from scratch using BertTokenizerFast. BertTokenizerFast is a transformer model based on a tokenization algorithm that splits words into meaningful subwords. The algorithm thus can capture semantic meaning in agglutinative languages such as Turkish, where complex words can be formed by combining several subwords. Upon training, BertTokenizerFast converts text input to a machine-readable numerical format based on a dictionary of the most frequently occurring 100,000 subwords. We use this self-trained BertTokenizerFast algorithm to tokenize Twitter features.

Step 3: Partitioning the sample

To avoid overfitting, we partition our sample into three subsets: training set, validation set, and test set. As is standard, we set aside 20% of the sample for validation and test sets and the remaining 80% for the training set. We use the training set to fit our model, the validation set to estimate the hyperparameters, and the test set to evaluate the predictive ability of the model. To choose the most suitable model with the highest "out-of-sample" accuracy, we compare the performance across validation and test samples. We rely on stratified sampling, as the number of users in different countries can be imbalanced and a random sampling strategy could bias the sampling toward bigger countries. We start with a relatively large training dataset (around 3.9 million tweets), which the prior literature deems sufficient for a high-quality model [5].

Step 4: Model Architecture

Next, we train an LSTM model based on the network architecture in [3]. In natural language processing, recurrent neural networks (RNNs) represent temporal sequences better than vanilla neural networks or fully connected layers do. LSTM networks are a specific type of RNN with memory cells enabling them to retain longer-range dependencies than conventional RNNs. We rely on the model in [3] to build a set of prediction models and estimate the model parameters in the training set. Supplementary Appendix Fig 1 illustrates the model architecture.

[Insert Supplementary Appendix
Fig 1 about here]

The model inputs tokenized text and represents it as word embeddings (i.e., vector representation with a lower number of dimensions). This provides advantages over one-hot encoding, which creates a sparse vector with a lack of context. Learned embeddings group words with similar locational semantics and thus improve efficiency. To avoid overfitting (and to penalize large coefficients), we use dropout layers [6] and randomly disable a proportion of neuron connections (or ignore some layer outputs). Batch normalization is used to reduce internal covariate shift and improve convergence speed during training [7]. The tanh activation function is used after the LSTM layer, and the softmax activation function is used to produce output logits (or probability scores for each class) after the final fully connected layer. We tune the hyperparameters of the model (i.e., learning rate, embedding dimension, and the number of

LSTM layers) based on evaluation metrics over the validation set. The performance of each model is assessed on the validation set to identify the parameters that yield the highest prediction accuracy.

Step 5: Tuning Model Parameters

The model is trained on a single NVIDIA GeForce RTX 3090 GPU, with a batch size of 64. We choose the Adam optimization algorithm as an extension of stochastic gradient descent, which results in a stable and faster convergence [8]. The dataset is trained for five epochs, which corresponds to one pass through the whole training dataset. Since we have a large training set with about 3.9 million entries, the training losses and accuracies saturate within five epochs. The learning rate is an important parameter, as it determines the step size of the iterative improvement in stochastic gradient descent. We perform a hyperparameter search on the learning rates at 1e-2, 1e-3, 1e-4, and we report the results in Panel A of Supplementary Appendix Table 1.

[Insert Supplementary Appendix
Table 1 about here]

We also vary the parameters (as shown in Panel B) to choose the set of optimal hyperparameters maximizing the predictive ability in the validation set. The optimized model in [3] uses word-embedding dimensions of 100 and one LSTM layer. As we use a different training set, we implement two modifications: (i) the word-embedding dimension is increased from the original 100 to 250, and (ii) the number of LSTM layers is increased from one to three. We perform these experiments to determine the optimal set of parameters in our dataset. When evaluating the models, we consider two performance metrics. We first use *Accuracy*, defined as the ratio of correct location predictions, as follows:

$$Acc = \frac{|\{t \in \mathrm{T}: c(t) = c^*(t)\}|}{|T|}$$

where $c(t)$ and $c^*(t)$ represent the predicted and ground-truth locations for tweet t. Second, we calculate the mean error distance (*Mean-ED*) as the Haversine distance between the predicted and ground-truth location. The model with the lower *Mean-ED* and higher *Accuracy* is better at predicting the geolocation of tweets. We present the results in Supplementary Appendix Table 1, Panel B.

We choose the parameters with the lowest *Mean-ED* and highest *Accuracy*. Accordingly, we use an initial learning rate of 1e-3, a word-embedding dimension of 100, and two LSTM layers in the feed-forward model. As learning progresses, the parameters in the LSTM are adjusted via backpropagation so the network continually improves its ability to predict a tweet's geolocation. We connect the LSTM layer with a dense layer for classification using a softmax activation function. Our final model estimates 15,090,237 parameters.

Supplementary Appendix 2

Conversion of fact-checking categories to fake and non-fake news

Each fact-checking organization has its own news classification scheme. We map these categories to fake and non-fake news as follows.

a) *Snopes*: “False”, “mostly false”, “misattributed”, “miscaptioned”, “not quite”, “probably false”, and “scam” are classified as fake news. “True”, “correct attribution”, “mostly true”, and “sort of” are classified as non-fake news.

b) *Politifact*: “False”, “barely-true”, and “pants-on-fire” are classified as fake news. “True” and “mostly true” are classified as non-fake news.

c) *Truthorfiction*: “False”, “misleading”, “fiction”, “not true”, and “decontextualized” are classified as fake news. “True”, “truth”, “truth, previously truth” and “truth, previously truth, now ended” are classified as non-fake news.

d) *Factcheck*: There is no classification scheme. We manually read articles and determine the claim’s veracity. The results are robust to the exclusion of articles obtained from this site.

Supplementary Appendix 3

Final labeling rules for identifying firm-related news

We agreed upon the following labeling rules to identify firm-related news:

1. The claim should be directly about the firm; just mentioning a firm (or a product) name in the text is not sufficient.

(a) Discussion of a generic product is not sufficient if the product is not linked to a specific firm (see, e.g., https://www.snopes.com/fact-check/breastfeeding-baby-covid-vaccine/).

(b) Discussion of a generic product is sufficient if the product is linked to a specific firm (see, e.g., https://www.snopes.com/fact-check/dr-rajendra-kapila-dies-covid-19/).

(c) If the claim about a person is not directly related to the firm employing the person, do not categorize the news as firm related (see, e.g., https://www.snopes.com/fact-check/amber-heard-fired/).

(d) If a firm (or a product) is mentioned as part of a story line, do not categorize the news as firm related (see, e.g., https://www.snopes.com/fact-check/1860-painting-woman-iphone/ or https://www.snopes.com/fact-check/mcdonalds-countries-war/).

(e) If the claim is about the operations of a firm, categorize the news as firm related (see, e.g., https://www.snopes.com/fact-check/splash-mountain-disneyland/, https://www.snopes.com/fact-check/apple-emergency-broadcast-system/ or https://www.snopes.com/fact-check/did-abcs-vp-programming-cancel-roseanne/).

2. The mere fact that a claim is circulating on a particular social media platform does not, by itself, mean that the news is related to that particular social media firm.

(a) If a rumor takes place in Facebook but is not directly about Facebook (or other firms), do not categorize it as firm related (e.g., https://www.snopes.com/fact-check/bora-bora-getaway-facebook/).

(b) If the claim is about, for example, the number of followers of a Twitter account but is not directly about Twitter, do not categorize it as firm related (see, e.g., https://www.snopes.com/fact-check/trump-lose-followers-first-time/).

(c) In contrast, if the news is directly about the operations of a social media platform, categorize it as firm related (see, e.g., https://www.snopes.com/fact-check/trump-banned-for-life-facebook/ or https://www.snopes.com/fact-check/paypal-twitter-jar-address/).

3. An event may take place on the premises of a firm, but if the firm (or its employees) is not involved with the incident, do not link the news to the firm.

(a) If a hate crime is committed on the premises of a firm, but the firm's employees are not involved, do not categorize the news as firm related (see, e.g., https://www.snopes.com/fact-check/muslim-woman-told-to-hang-herself-with-hijab-at-walmart/).

(b) In contrast, if the news is about an intentional corporate policy, categorize the news as firm related (see, e.g., https://www.snopes.com/fact-check/walmart-audio-blm/).

4. News about the founder/executive of a firm is not necessarily firm related.

(a) If the news is about the political ideology of a firm's founders/executives or their charitable activities, do not categorize the news as firm related (see, e.g., https://www.snopes.com/fact-check/bill-gates-planned-parenthood/, https://www.truthorfiction.com/ellison-yale/ or https://www.politifact.com/factchecks/2020/may/14/facebook-posts/no-evidence-gates-foundation-will-profit-coronavir/).

(b) The arrest or death of a firm's founders/executives, however, may affect the operations of a firm and therefore should be categorized as firm related (see, e.g., https://www.snopes.com/fact-check/italy-bill-gates-arrest/ or https://www.snopes.com/fact-check/elon-musk-death-hoax/).

5. Do not categorize politicians' statements about a firm as firm related (see, e.g., https://www.politifact.com/factchecks/2011/feb/09/jim-renacci/rep-jim-renaccis-gm-stock-claim-crashes-accuracy/).

6. If the news encompasses several firms in an industry but is not about a specific firm, do not categorize it as firm related (see, e.g., https://www.snopes.com/fact-check/stores-locking-tide-pods-people-wont-eat/).

7. In case of doubt and in all other cases, the claim must be labeled as non-firm related.

Supplementary Appendix 4
Bot activity check

We use Botometer as a machine learning–based public tool for bot detection on Twitter [9]. Botometer is a popular tool that has been extensively used and validated in the literature. It uses a combination of random forest classifiers and exploits over 1,000 features, including user profile, network, content, language, and sentiment to capture bot-like behavior (e.g., length of the screen name, the age of the account, and the number of verbs, nouns, and adjectives in the tweets).

To train the model, the authors of [9] use a variety of annotated datasets comprising both bot and human accounts (https://botometer.osome.iu.edu/bot-repository/). The training data consist of over 90,000 bot accounts and 40,000 human accounts [10]. After experimenting with several models, the authors of [9] choose the one with the highest predictive accuracy (which achieves an AUC (area under the curve) of 0.99). The model is also constantly upgraded (using new training data and feature updates) to keep up with the evolving behavior of bot and human accounts.

The tool provides a bot score (called the complete automation probability) ranging from 0 to 1, with higher scores indicating a higher likelihood that a Twitter account is a bot (https://botometer.osome.iu.edu/). A Twitter account with a score greater than 0.50 is considered more likely to exhibit bot characteristics [11]. We subscribe to the Botometer Pro API (Ultra) account to programmatically check accounts in bulk (https://rapidapi.com/OSoMe/api/botometer-pro/pricing).

Supplementary Appendix 5

Variable definitions

Variable	Description	Data source
News Data		
Fake News	Negative-sentiment news about a firm debunked by a fact-checking organization.	Fact-checking sites
Non-fake News	Negative-sentiment news about a firm verified by a fact-checking organization.	Fact-checking sites
News Content		
Data Privacy	An indicator variable equal to one if the news is related to data privacy (e.g., data breach, hacking, etc.), and zero otherwise.	Fact-checking sites
Founder/Executive Management	An indicator variable equal to one if the news is related to the firm's founders or executive management, and zero otherwise.	Fact-checking sites
Product	An indicator variable equal to one if the news is related to the firm's product (e.g., ingredients, spoilage, branding, etc.), and zero otherwise.	Fact-checking sites
Politics	An indicator variable equal to one if the news is related to politics (e.g., gun control, refugees, privatization, etc.), and zero otherwise.	Fact-checking sites
Religion	An indicator variable equal to one if the news is related to religion (e.g., religious belief, symbols, etc.), and zero otherwise.	Fact-checking sites
Operations	An indicator variable equal to one if the news is related to the firm's operations (e.g., investment, advertising, distribution, etc.), and zero otherwise.	Fact-checking sites
Other	An indicator variable equal to one if the news is related to corporate social responsibility, legal, or financial issues, and zero otherwise.	Fact-checking sites
Twitter Data		
Foreign Fake News (%)	Percentage of original tweets spreading corporate fake news initiated by a foreign (non-U.S.) Twitter account.	Twitter Academic API
Foreign Fake News (Dummy)	An indicator variable equal to one if at least one original tweet spreading corporate fake news is initiated by a foreign (non-U.S.) Twitter account, and zero otherwise.	Twitter Academic API
Foreign Non-fake News (%)	Percentage of original tweets spreading corporate non-fake news initiated by a foreign (non-U.S.) Twitter account.	Twitter Academic API
Foreign Non-fake News (Dummy)	An indicator variable equal to one if at least one original tweet spreading corporate non-fake news is initiated by a foreign (non-U.S.) Twitter account, and zero otherwise.	Twitter Academic API
Bot Score	A score between zero and one used to measure the bot-like activity of a Twitter account. A higher score indicates a higher probability that the account is a bot.	Botometer API
# Followers	Number of people who follow the user on Twitter.	Twitter Academic API

Supplementary Appendix 5

(*continued*)

Variable	Description	Data source
# Followees	Number of people the user follows on Twitter.	Twitter Academic API
Account Age	The age of the user's account, measured in years.	Twitter Academic API
Information Environment		
Institutional Ownership	Percentage of the target company's stock held by institutional investors as of the beginning of the quarter in which the fake news is published.	Thomson Reuters 13F
Return Volatility	Standard deviation of a firm's daily stock returns during a fiscal year.	CRSP
Foreign Sales	An indicator variable equal to one if at least 10% of a firm's sales are to foreign markets.	COMPUSTAT
Strategic Importance		
Strategic Industry	An indicator variable equal to one if the firm is in the telecommunication, pharmaceutical, semiconductor, computer, or defense industry (i.e., SIC = 4812-4899, 3663, 2833-2836, 3674, 3570-3577, 7370, 7372, 9711).	COMPUSTAT
Industry Leader	An indicator variable equal to one if the firm is the largest member of its industry in terms of revenue, and zero otherwise.	COMPUSTAT
Market Structure		
TNIC HHI	Sum of the squares of the sales (SALE) of firms in the same industry, using the time-varying Text-based Network Industry Classification (TNIC) developed by Hoberg and Phillips [12].	Hoberg-Phillips Data Library (http://hobergphillips.tuck.dartmouth.edu)
Product Similarity	A firm-year-level measure of product similarity based on the product descriptions from 10-K filings calculated as the sum of pairwise product similarities between a given firm and all other firms in a given year [12].	Hoberg-Phillips Data Library (http://hobergphillips.tuck.dartmouth.edu)
Geopolitical Risks		
Interstate Conflict Risk	Index measuring interstate conflict risk (based on the Goldstein scale) using daily reported events in the global news media.	Global Database on Event, Location, and Tone (GDELT)
Geopolitical Risk Index	Index based on the share of articles mentioning adverse geopolitical events in leading newspapers in the U.S.	Caldara and Iacoviello [13]

Supplementary Appendix 5

(*continued*)

Variable	Description	Data source
Control Variables		
Total Assets	Log of total assets (AT).	COMPUSTAT
Book-to-market	Log of Common Equity (CEQ + TXDITC – PS) /Market Capitalization (CSHO*PRCC_F) ratio.	COMPUSTAT
LEV	Sum of long-term debt (DLTT) and short-term debt (DLC) divided by total assets (AT).	COMPUSTAT
RNOA	Ratio (in percentage) of operating income to average net operating assets (Net Accounts Receivable + Inventories + All Other Current Assets + Net PPE + Intangibles + All Other Assets - Accounts Payable - All Other Current Liabilities - All Other Liabilities).	COMPUSTAT
Div	Dividends (DVPSX_F) divided by fiscal year-end price (PRCC_F).	COMPUSTAT
Loss	An indicator variable equal to one if a firm reports negative earnings.	COMPUSTAT

Supplementary Appendix Figure 1
LSTM model architecture

This figure illustrates the model architecture of the location-prediction model. The model begins by inputting tokenized text and representing it as word embeddings (i.e., vector representation with a lower number of dimensions). Learned embeddings group words with similar locational semantics and improve efficiency. Next, we use dropout layers and randomly disable a proportion of neuron connections to avoid overfitting (and to penalize large coefficients). Batch normalization is then used to reduce internal covariate shift and improve convergence speed during training. After the second dropout, we use the tanh activation function after the LSTM layer. After repeating batch normalization and dropout, we use the softmax activation function to produce output logits (or probability scores for each class) after the final fully connected layer.

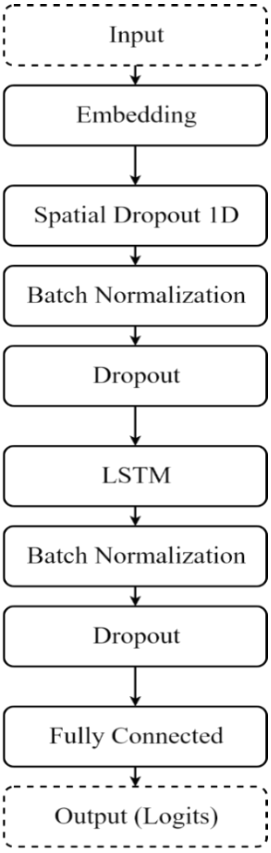

Supplementary Appendix Table 1
Hyperparameter search

This table shows the hyperparameter search of the model. We tune the model's hyperparameters (i.e., learning rate, embedding dimension and the number of LSTM layers) based on evaluation metrics over the validation set. We use two evaluation metrics. First, we use mean error distance (*Mean-ED*) as the average distance in kilometers between the model prediction and ground-truth location at the city level. Second, we use accuracy (*Acc*) as the proportion of model predictions that correctly predict ground-truth country location at the country level. The performance of each model is assessed on the validation set to identify the parameters that yield the highest prediction accuracy. Panel A reports the evaluation metrics for the initial learning rate. Panel B reports the evaluation metrics for the embedding dimensions and LSTM layers.

Panel A. Initial Learning Rate

Initial LR	*Mean-ED*	*Acc*
1e-2	1545.48	81.01
**1e-3**	**908.33**	**88.76**
1e-4	986.24	87.81

Panel B. Model Hyperparameters

Embedding Dimension/ #LSTM	*1*	***2***	*3*
100	88.51 (924.85)	**88.76 (908.33)**	88.46 (926.50)
150	88.36 (944.19)	88.58 (919.80)	88.54 (916.12)
200	88.43 (927.81)	88.65 (917.54)	88.63 (911.65)
250	88.28 (941.87)	88.58 (913.89)	88.40 (938.86)

References

1. Zubiaga A, Voss A, Procter R, Liakata M, Wang B, Tsakalidis A. Towards real-time, country-level location classification of worldwide tweets. IEEE Transactions on Knowledge and Data Engineering. 2017; 29(9): 2053-2066.
2. Kruspe A, Häberle M, Hoffmann EJ, Rode-Hasinger S, Abdulahhad K, Zhu XX. Changes in Twitter geolocations: insights and suggestions for future usage. 2021. Available from: https://arxiv.org/abs/2108.12251.
3. Thomas P, Hennig L. Twitter geolocation prediction using neural networks. Language Technologies for the Challenges of the Digital Age: 27th International Conference Proceedings. 2018; 248-255.
4. Zheng X, Han J, Sun A. A survey of location prediction on Twitter. IEEE Transactions on Knowledge and Data Engineering. 2018; 30(9): 1652-1671.
5. Han B, Cook P, Baldwin T. Text-based Twitter user geolocation prediction. Journal of Artificial Intelligence Research. 2014; 49: 451-500.
6. Hinton GE, Srivastava N, Krizhevsky A, Sutskever I, Salakhutdinov RR. Improving neural networks by preventing co-adaptation of feature detectors. 2012. Available from: https://doi.org/10.48550/arXiv.1207.0580.
7. Ioffe S, Szegedy C. Batch Normalization: Accelerating Deep Network Training by Reducing Internal Covariate Shift. 2015. Available from: https://doi.org/10.48550 /arXiv.1502.03167.
8. Kingma DP, Ba J. Adam: A Method for Stochastic Optimization. 2017. Available from: https://doi.org/10.48550/arXiv.1412.6980.
9. Yang KC, Ferrara E, Menczer F. Botometer 101: Social bot practicum for computational social scientists. Journal of Computational Social Science. 2022; 5: 1-18.
10. Yang KC, Varol O, Hui PM, Menczer F. Scalable and generalizable social bot detection through data selection. Proceedings of the AAAI Conference on Artificial Intelligence. 2020; 34: 1096-1103.
11. Shao C, Ciampaglia GL, Varol O, Yang KC, Flammini A, Menczer F. The spread of low-credibility content by social bots. Nature Communications. 2018; 9(1): 1-9.
12. Hoberg G, Phillips G. Text-based network industries and endogenous product differentiation. Journal of Political Economy. 2016; 124(5): 1423-1465.
13. Caldara D, Iacoviello M. Measuring geopolitical risk. American Economic Review. 2022; 112(4): 1194-1225.
